# Alkylating Agent–Induced FANCL Impairment Damages Pulmonary Arterial, Venous, and Microvascular Endothelial Cells and Predisposes to PAH/PVOD

**DOI:** 10.1002/advs.78081

**Published:** 2026-10-08

**Authors:** Shiyun Liu, Yufei Sun, Xinlei Wu, Haixia Chen, Shaocong Mo, Zichong Li, Yiwei Gong, Xiaoqian Shan, Yuanqi Li, Yaru Guan, Junqi Zhu, Han Yan, Min Du, Feng Wei, Peixin Ou, Ziyi Wang, Chenting Zhang, Lei Xu, Wenju Lu, Jian Wang, Kai Yang

**Affiliations:** ^1^ The Fifth Affiliated Hospital of Guangzhou Medical University Guangzhou Guangdong China; ^2^ State Key Laboratory of Respiratory Disease National Clinical Research Center for Respiratory Disease National Center for Respiratory Medicine Guangdong Basic Research Center of Excellence for Respiratory Medicine Guangdong Key Laboratory of Vascular Disease Guangzhou Institute of Respiratory Health The First Affiliated Hospital of Guangzhou Medical University Guangzhou Guangdong China; ^3^ Department of Pathology Foshan Sanshui District People's Hospital Foshan Guangdong China; ^4^ School of Basic Medical Sciences Guangzhou Medical University Guangzhou Guangdong China; ^5^ Department of Pulmonary and Critical Care Medicine The Affiliated Hospital of Inner Mongolia Medical University Hohhot, Inner Mongolia Autonomous Region China; ^6^ School of Clinical Medicine Jilin Medical University Jilin China; ^7^ Guangzhou National Laboratory Guangzhou International Bio Island Guangzhou Guangdong China; ^8^ GMU‐GIBH Joint School of Life Sciences Guangzhou Medical University Guangzhou Guangdong China

**Keywords:** apoptosis, cisplatin, DNA damage, endothelial cell, fanconi anemia complementation group L, pulmonary arterial hypertension, pulmonary veno‐occlusive disease

## Abstract

Alkylating chemotherapies such as mitomycin C (MMC) and cisplatin (CDDP) are associated with pulmonary arterial hypertension (PAH) with pulmonary veno‐occlusive disease (PVOD) features, yet the underlying mechanisms remain unclear. Using established MMC and newly developed CDDP rat models, we found that both agents induced PAH/PVOD, as evidenced by elevated right ventricular systolic pressure, pulmonary arterial, venous and microvascular remodeling, and right ventricular dysfunction, with MMC causing more severe pathology. In cultured pulmonary arterial, venous, and microvascular endothelial cells, MMC and CDDP induced elevated DNA damage, apoptosis, and endothelial‐to‐mesenchymal transition, together with impaired angiogenic capacity, proliferation, and barrier junction. These phenotypical changes were accompanied by conserved Fanconi anemia complementation group L (FANCL) downregulation. FANCL deficiency in mice drove spontaneous and exacerbated CDDP‑induced PAH/PVOD, whereas AAV‑mediated FANCL rescue or treatment with a DNA protector, amifostine, significantly attenuated the disease pathogenesis in mouse and rat models. Collectively, this study establishes a novel CDDP‑induced rat model, identifies a conserved FANCL–DNA damage–endothelial dysfunction mechanistic axis underlying PAH/PVOD induced by MMC or CDDP, and highlights FANCL rescue and DNA damage inhibition as potential therapeutic strategies for this severe form of pulmonary vascular disease.

## Graphical abstract

This study establishes a novel cisplatin‐induced rat model of PAH/PVOD and identifies a conserved mechanistic axis—FANCL impairment, DNA damage accumulation, and endothelial dysfunction—triggered by cisplatin or mitomycin C across pulmonary arteries, veins, and microvessels. Importantly, FANCL restoration or DNA damage inhibition by amifostine effectively attenuates cisplatin‐induced PAH/PVOD pathogenesis, highlighting their therapeutic potential for this severe PAH subtype.

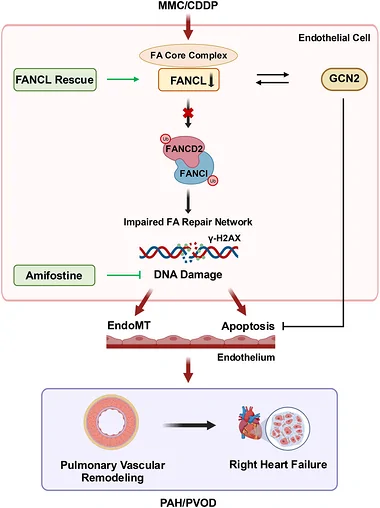

## 1 Introduction

Pulmonary arterial hypertension (PAH) is classified as Group 1 pulmonary hypertension (PH), characterized by increased pulmonary vascular resistance, plexiform lesions, arteriolar obliteration, and occlusive arterial remodeling [1]. Concentric pulmonary vascular remodeling, a key feature, manifests as vascular wall thickening and obliterative intimal lesions. These changes result from dysregulation of smooth muscle cells, endothelial cells, and fibroblasts [2, 3, 4]. According to the 2022 ESC/ERS guidelines for the diagnosis and treatment of PH and the 7th World Symposium on Pulmonary Hypertension (WSPH), pulmonary veno‐occlusive disease/pulmonary capillary hemangiomatosis (PVOD/PCH) is classified as the subgroup 1.5 of Group 1 PAH [1, 5, 6, 7, 8, 9, 10]. PAH primarily involves precapillary pulmonary arterioles with plexiform lesions, whereas PVOD is characterized by fibrous intimal proliferation of septal veins and preseptal venules, often accompanied by pulmonary capillary dilatation and proliferation (pulmonary capillary hemangiomatosis‐like changes). Current understanding of this PAH with venous/capillary (PVOD/PCH) involvement remains limited. There is an urgent need to elucidate the disease pathology and to develop novel, effective therapeutic strategies for PAH/PVOD.

Alkylating chemotherapeutic agents induce DNA interstrand cross‐links (ICLs) to damage cancer cells [11]. In mammalian cells, the accumulation of DNA damage, resulting from endogenous or exogenous sources and/or insufficient repair, leads to genomic instability, ultimately triggering cell death, senescence, or phenotypic changes [12]. Clinical observations have reported that patients can develop PAH/PVOD following exposure to alkylating chemotherapeutic agents such as Mitomycin C (MMC), Cisplatin (CDDP), or Cyclophosphamide (CP) [13]. According to the 7th WSPH clinical classification, MMC has been reported to be associated with PAH with features of PVOD/PCH involvement [7]. A series of studies in rodent and rabbit models have established a causal link between alkylating agent exposure and the development of PAH/PVOD. These models faithfully recapitulate the characteristic pulmonary microvascular remodeling and occlusion—affecting small veins and capillaries—that are hallmarks of the human disease [13, 14, 15]. Neubert et al. [16] reported that MMC exposure in rats primarily induces arterial and microvascular remodeling, with venous remodeling becoming evident predominantly when PVOD symptoms manifest. Complementing these findings, our previous studies demonstrated that the apoptosis and endothelial‐to‐mesenchymal transition (EndoMT) in pulmonary microvascular endothelial cells (PMVECs) are common features within the pulmonary microvasculature (encompassing arteries and veins) of MMC‐induced PAH/PVOD rats [15] and mice [17].

Based on previous findings, this study systematically investigates the time‐dependent effects of two commonly used alkylating chemotherapeutic agents, MMC and CDDP, on cardiopulmonary hemodynamics and pathology. While MMC exposure has been reported to induce PAH/PVOD in rats [14, 15], the pathogenic role of CDDP in PAH/PVOD was investigated through comprehensive assessments of the hemodynamic and pathological changes in the lung, alongside histological and functional alterations in the heart. Additionally, using freshly isolated rat PMVECs and primary cultured endothelial cells from pulmonary arteries (PAECs), veins (PVECs), and microvessels (PMVECs), the morphological and functional alterations induced by MMC or CDDP were assessed. These data provide essential preclinical evidence, establishing a novel rat model and offering therapeutic targets for alkylating agent‑induced PAH/PVOD.

## 2 Materials and Methods

The extended methods are provided in the online Supporting Information.

### 2.1 Chemicals and Reagents

The commercial sources of major reagents and chemicals include: Mitomycin C (MMC, S8146, Selleck, Shanghai, China), Cisplatin (CDDP, MB1055, Meilunbio, Dalian, China), SU5416 (HY‐10374, MedChemExpress, Monmouth Junction, NJ, USA), Amifostine trihydrate (S4298, Selleck, Shanghai, China). Primary antibodies for fibronectin (FN, #15613‐I‐AP, Proteintech, Rosemont, IL, USA), Twist (ab50887, Abcam, Cambridge, MA, USA), Vimentin (#5741, Cell Signaling Technology, Danvers, MA, USA), Snail (#ab53519, Abcam, Cambridge, MA, USA), ZO‐1 (21773‐1‐AP, proteintech, Wuhan, China), VEGFR2 (9698, CST, Boston, USA), Slug (YM8357, Immunoway, California, USA), proliferating cell nuclear antigen (PCNA, ab29, Abcam, Cambridge, MA, USA), CD31 (AF3628, R&D Systems, Minneapolis, MN, USA), Cleaved caspase3 (#9664, Cell Signaling Technology, Danvers, MA, USA), Bax (#2772S, Cell Signaling Technology, Danvers, MA, USA), B‐cell lymphoma/leukemia 2 (Bcl‐2, 13–8800, Thermo Fisher Scientific, Rockford, IL, USA), γ‐H2AX (#05‐635, EMD Millipore/Merck, USA), α‐smooth muscle actin (α‐SMA, ab124964 and ab7817, Abcam, Cambridge, MA, USA), FANCL (LS‐C807939, LifeSpan BioSciences, Seattle, WA, USA), FANCD2 (NB100‐182, Novus Biologicals, USA), general control nonderepressible 2 (GCN2, #46041, Cell Signaling Technology, Danvers, MA, USA), and β‐actin (66009‐1‐Ig, Proteintech, Rosemont, IL, USA) were used for immunoblotting, immunofluorescence (IF), and immunohistochemistry (IHC) staining.

### 2.2 Animal Ethics and Procedures

All animal procedures complied with the ARRIVE guidelines and were approved by the Animal Care and Use Committee of The First Affiliated Hospital of Guangzhou Medical University (Approval Number: 2020–86) and Guangzhou Medical University (Approval Number: GY2026‐0886 and GY2026‐0782). All procedures conformed to the NIH Guide for the Care and Use of Laboratory Animals. Only female rats were used based on the previous report of greater sensitivity to MMC‑induced pulmonary vascular toxicity, particularly with respect to venous remodeling, compared with male rats [14]. Adult female Sprague‐Dawley (SD) rats (body weight: 150–200 g) were obtained from Charles River Laboratories Inc. (Hangzhou and Guangzhou, China). Rats were housed in a Specific Pathogen Free (SPF) grade animal facility at The First Affiliated Hospital of Guangzhou Medical University. All procedures were conducted with the utmost care to ensure animal welfare and minimize suffering, adhering to the principles of the 3Rs (Replacement, Reduction, Refinement). The experimental procedures and data analysis were performed in a blinded manner.

#### 2.2.1 Rat Models

*Protocol 1*: Rats were randomly assigned to treatment groups and received intraperitoneal (i.p.) injections of either MMC (3 mg/kg) or CDDP (4 mg/kg) once per week for two weeks. They were then harvested at 2 or 4 weeks after the initial administration. Prior to terminal procedures, rats were anesthetized with a single dose of sodium pentobarbital (50 mg/kg, i.p.) and subjected to hemodynamic assessments. Following the hemodynamic measurements, the rats were euthanized by exsanguination under deep anesthesia. The heart and lungs were then excised for subsequent histological and molecular biological analysis.

*Protocol 2*: For the separate amifostine intervention study, rats were assigned to four groups: control (saline), CDDP alone, amifostine alone, and CDDP + amifostine. CDDP (4 mg/kg, i.p.) was given on days 0 and 7. Amifostine (150 mg/kg, i.p.) was administered 20 min before each CDDP injection in the combination group; amifostine ‐alone rats received amifostine on the same schedule without CDDP, and control rats received saline. All animals were euthanized on day 28, and target tissues were harvested.

*Protocol 3*: For the establishment of Sugen/Hypoxia (SuHx)‐induced PAH rat model, rats received a single injection of SU5416 (20 mg/kg, i.p.) and were subsequently exposed to chronic hypoxia (10% O_2_) for 3 weeks, followed by a 3‐week recovery period under normoxia in accordance with established protocols routinely used in our laboratory [18]. Successful induction of PAH was confirmed by characteristic hemodynamic and histological parameters.

#### 2.2.2 Mouse Model

*Fancl* ^kf/kf^ mice on a C57BL/6N background were generated by the Nanjing Biomedical Research Institute, Nanjing University, as described previously [19]. For AAV‐mediated gene delivery, wild‐type (WT) and *Fancl* ^kf/kf^ mice were divided into five groups: WT, WT + CDDP, *Fancl* ^kf/kf^, *Fancl* ^kf/kf^ + CDDP + AAV‐Con, and *Fancl* ^kf/kf^ + CDDP + AAV‐*Fancl*. On day 7, mice received a single intratracheal instillation of adeno‐associated virus 9 (AAV9) particles (1 × 10^11^ genome copies in 50 µL PBS; PackGene Biotech, Guangzhou, China) encoding *Fancl* (AAV‐*Fancl*) or control (AAV‐Con); the remaining groups received PBS vehicle. CDDP was then administered i.p. at 3 mg/kg on days 0, 3, 6, 9, 12 and 15 (six doses total); control mice received saline. All mice were euthanized on day 42, and lung tissues were collected.

### 2.3 Hemodynamic Assessment

The right ventricular systolic pressure (RVSP) was measured by a BIOPAC MP150 data acquisition system (BIOPAC systems, Inc., Santa Barbara, CA). The right ventricular hypertrophy was assessed by the Fulton Index, calculated by the mass ratio of right ventricle to left ventricle plus septum [RV/(LV+S)]. These procedures were all conducted according to our previously established procedures [15, 18].

### 2.4 Transthoracic Echocardiography

After each treatment, rats were anesthetized by inhaled isoflurane (1.5%) through a facemask and transthoracic echocardiographic assessments were performed using a VisualSonics Vevo 2100 system (VisualSonics Inc., Toronto, ON, Canada) and a transducer (MS‐400, 20–46 MHz). The heart function was measured by echocardiography analysis of specific indexes including the pulmonary acceleration time (PAT) and pulmonary ejection time (PET), changes in RV fractional area (RV FAC), tricuspid annular plane systolic excursion (TAPSE), right ventricular end‐diastolic wall thickness (RVEDWT), right ventricular end‐systolic wall thickness (RVESWT), ejection fraction (EF), cardiac output (CO) and left ventricular fractional shortening (LVFS).

### 2.5 Transfection of Small Interfering RNA (siRNA)

Cells were transfected with siRNA using Lipofectamine^TM^ 3000 Reagent (Invitrogen, USA) according to the manufacturer's instructions. Cells were collected and harvested for further experiments including immunoblotting, IF staining, comet assay, and cell apoptosis assessments. The siRNA for *Fancd2* and *Fancl* were purchased from GenePharma (Shanghai, China) and *Eif2ak4* from Thermo Fisher Scientific (Rockford, USA), and the sequences are listed in Table S1.

### 2.6 Statistical Analysis

Data are expressed as mean ± SD. The sample size (n) represents the number of animals or independent cell culture experiments, as specified in the respective figure legends. Normality (Shapiro‐Wilk test) and homogeneity of variance (Bartlett's test) were assessed prior to further analysis. For data that passed the tests of normal distribution and homogeneity, differences between two groups were assessed by *t*‐test, and differences for three or more groups were assessed by one‐way ANOVA followed by Tukey's honestly significant difference (Tukey's HSD) as a post hoc test for multiple comparison test, or two‐way ANOVA followed by a Tukey's HSD post hoc test for multiple comparisons (with an interaction *P* value described in the legend of each figure). For data that did not pass the tests of normal distribution or homogeneity, a non‐parametric Mann‐Whitney U‐test was used for pairwise comparison between two groups. A *P* value < 0.05 is considered statistically significant. GraphPad Prism 7 and SPSS 23.0 were used for statistical analyses. The experiments were analyzed by an investigator blinded to the grouping and treatments.

## 3 Results

### 3.1 Exposure to Alkylating Agents MMC or CDDP Induced Progressive PAH in Rats

Adult female rats were randomly assigned to groups and treated with MMC or CDDP for 2 or 4 weeks. Results for the MMC‐induced PAH rat model were generally consistent with previous reports [14, 15]. MMC treatment induced a significant decline in body weight (Figure 1A) and survival rate (Figure 1B). The significant body weight loss in MMC‑treated rats is a well‑recognized manifestation of its systemic cytotoxicity, primarily driven by gastrointestinal toxicity [20, 21] and also attributed by myelosuppression, renal dysfunction, and reproductive organ toxicity [22, 23]. Furthermore, it led to progressive increases in right ventricular systolic pressure (RVSP, Figure 1C,D) and right ventricular hypertrophy, as reflected by elevated Fulton Index [RV/(LV+S), Figure 1E] and RV/body weight ratio (Figure 1F). H&E staining and Weigert's resorcin‑fuchsin staining for elastin fibers were performed in combination on sequentially cut lung sections to distinguish distal pulmonary arteries (PAs, characterized by two layers of elastic fibers), pulmonary veins (PVs, one layer), and microvessels/capillaries (PMVs, defined as extremely thin‑walled vessels with a diameter below 20 µm), indicating excessive smooth muscle thickening, indicative of elevated pulmonary vascular remodeling (Figure 1G–I). Compared to MMC, CDDP exposure induced similar but less pronounced effects on body weight change (Figure 1J), survival rate (Figure 1K), RVSP (Figure 1L,M), Fulton Index (Figure 1N) and RV/body weight ratio (Figure 1O), as well as pulmonary microvascular remodeling (Figure 1P–R).

**FIGURE 1:**
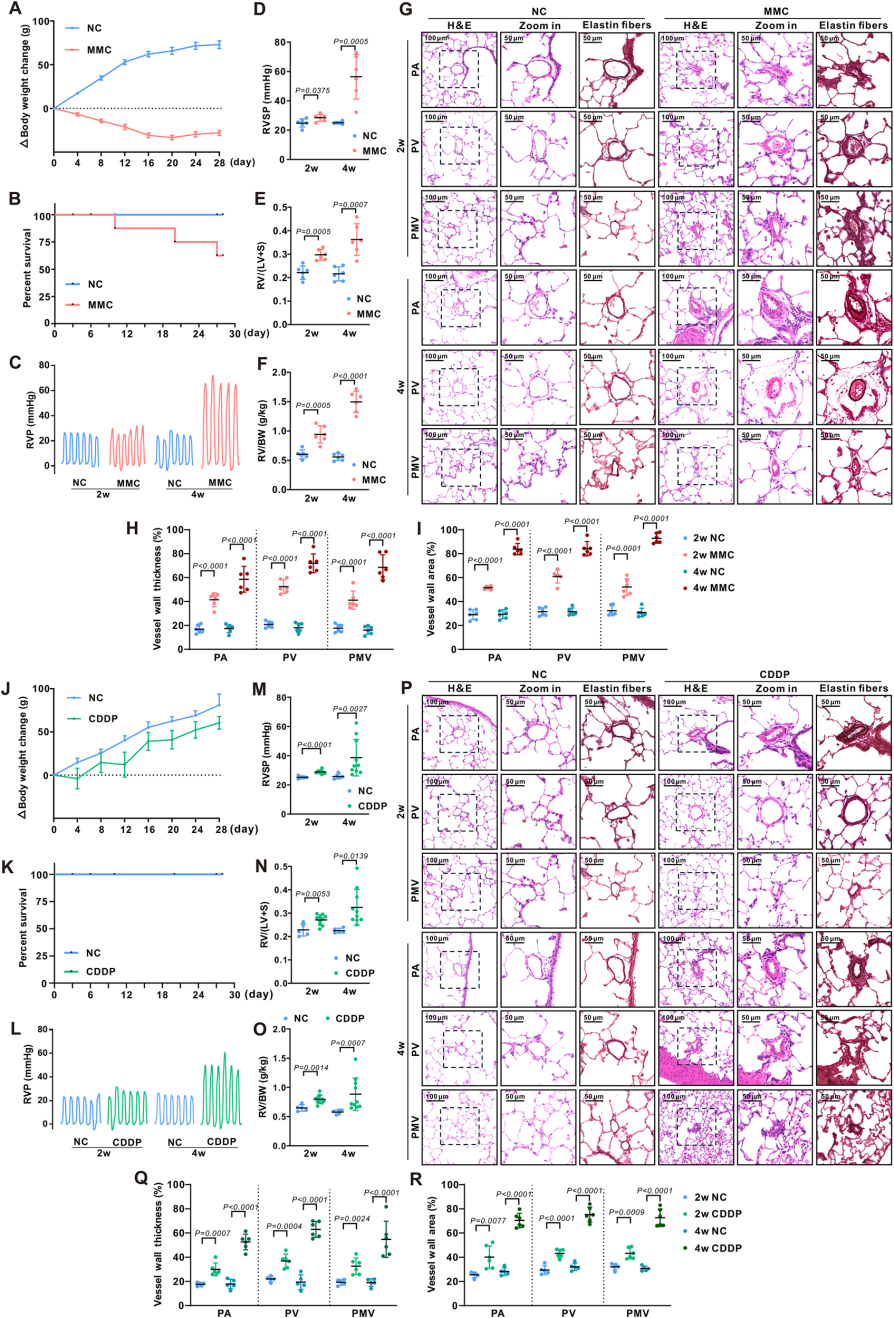
MMC or CDDP Treatment Induces Progressive PAH/PVOD in Rats. (A, B) Line graphs showing the time course survival rate and change of body weight in rats treated with MMC (3 mg/kg, once weekly) for 2 or 4 weeks. (C–F) Traces and summarized data showing the right ventricular systolic pressure (RVSP), Fulton Index [RV/(LV+S)], and RV/body weight (RV/BW) in rats treated with MMC (3 mg/kg, once weekly) for 2 or 4 weeks, *n* = 6 rats in each group. (G–I) H&E and Weigert's resorcin‐fuchsin staining and analyzed bar graphs showing the % vessel wall thickness and % vascular wall area for distal pulmonary arteries (PA), pulmonary veins (PV) and pulmonary microvessels (PMV) in rats treated with MMC (3 mg/kg, once weekly) for 2 or 4 weeks, *n* = 6 rats comprising around 30 vessels in each group. (J, K) Line graphs showing the time course survival rate and change of body weight in rats treated with CDDP (4 mg/kg, once weekly) for 2 or 4 weeks. (L–O) Traces and summarized data showing the RVSP, Fulton Index, and RV/BW in rats treated with CDDP (4 mg/kg, once weekly) for 2 or 4 weeks. For each time point (2 weeks and 4 weeks), *n* = 5 rats for control groups and *n* = 10 rats for CDDP‑treated groups. (P–R) H&E and Weigert's resorcin‐fuchsin staining and analyzed bar graphs showing the % vessel wall thickness and % vascular wall area for distal PA, PV and PMV in rats treated with CDDP (4 mg/kg, once weekly) for 2 or 4 weeks. For each time point (2 weeks and 4 weeks), *n* = 5 rats for control groups and *n* = 6 rats for CDDP‑treated groups, comprising around 30 vessels in each group. Graph values are mean ± SD. Differences between two groups were analyzed by *t*‐test, except for the 2‐week PMV vessel wall thickness comparison in H, the 2‐week PMV vascular wall area comparison in I, the 4‐week RVSP comparison in M, the 4‐week RV/BW comparison in O, and the 2‐week PA vessel wall thickness comparison in Q, for which the Mann–Whitney U‐test was used. A value of *P* < 0.05 is considered statistically significant, n.s. indicates no statistical significance. Scale bar = 100 and 50 µm in G and P. NC: normal control.

PAH is also characterized by structural and functional cardiac alterations. Echocardiographic analysis revealed progressive changes in cardiac function parameters in rats treated with MMC (Figure S1A) or CDDP (Figure S1D), including PAT/PET, % RV FAC, TAPSE, RVEDWT, RVESWT, CO, % EF, and % FS. Additionally, H&E staining and Masson's trichrome staining of right ventricular sections demonstrated increasing cardiomyocytes hypertrophy and fibrosis in rats treated with MMC (Figure S1B,C) or CDDP (Figure S1E,F) for 2 and 4 weeks.

We also conducted a separate animal study to evaluate the potential acute effects of MMC and CDDP following short‑term administration (2‑day and 5‑day regimens). Under these conditions, neither RVSP (Figure S2A) nor histological parameters, including distal pulmonary vascular wall thickness (Figure S2B,C) and right ventricular hypertrophy (Figure S2D,E), were significantly altered. Immunohistochemistry (Figure S2F) and immunoblotting (Figure S2G,H) further revealed no significant changes in the apoptotic marker cleaved caspase3. However, given the significant right ventricular hypertrophy observed as early as 2 weeks in both MMC‑ and CDDP‑treated groups, together with previous reports of MMC/CDDP‑related cardiac toxicity, indicates the RV remodeling may be independent of—or at least not strictly secondary to—the pulmonary vascular pathology induced by MMC or CDDP.

The effects of MMC or CDDP on pulmonary vascular phenotypic changes were assessed by immunoblotting analysis of lung tissue lysates. Exposure to MMC (Figure 2A–D) or CDDP (Figure 2E–H) for 2 or 4 weeks progressively increased the levels of proteins associated with apoptosis signaling (cleaved caspase3 and Bax/Bcl2 ratio) and proliferation signaling (PCNA). Concurrently, both agents upregulated the mesenchymal marker fibronectin (FN) and the pro‑EndoMT transcription factor Twist, while downregulating the endothelial marker CD31.

**FIGURE 2:**
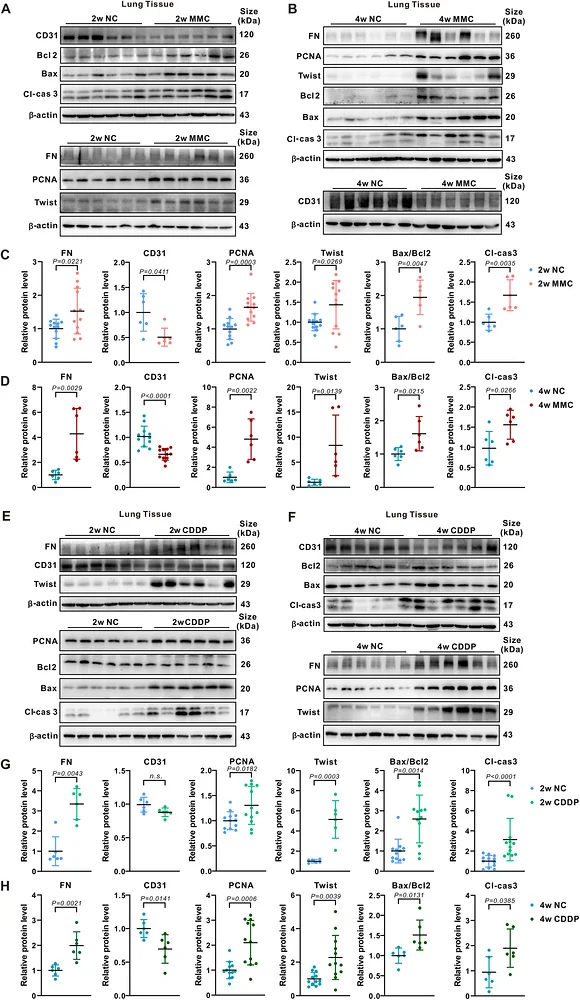
Effects of MMC and CDDP on Pulmonary Vascular Remodeling Marker Expression in Rat Lung Tissue. (A–H) Immunoblots and analyzed graphs showing the expression of proteins involved in the signaling pathways including apoptosis (cleaved caspase3, ratio of Bax/Bcl2) and proliferation (PCNA), pro‐EndoMT transcription factor Twist, mesenchymal cell marker FN, and endothelial cell marker CD31 in the lung tissue of rats treated with MMC (3 mg/kg, once weekly) for 2 or 4 weeks (A–D), and CDDP (4 mg/kg, once weekly) for 2 or 4 weeks (E–H). Data are presented as mean ± SD, *n* = 6–15 rats in each group. Protein expression levels were calculated as the ratio of the target protein band intensity to that of β‑actin and were normalized to the mean values of the respective 2‑week or 4‑week NC groups, expressed as fold change. Differences between two groups were analyzed by *t*‐test for C (FN, PCNA, Twist, Bax/Bcl2, Cl‐cas 3), D (FN, CD31, Twist, Bax/Bcl2, Cl‐cas 3), G (CD31, PCNA, Twist) and H (all panels), or Mann‐Whitney U‐test for C (CD31), D (PCNA) and G (FN, Bax/Bcl2, Cl‐cas 3). A value of *P* < 0.05 is considered statistically significant, n.s. indicates no statistical significance. NC: normal control.

### 3.2 Exposure to MMC or CDDP Induced Progressive DNA Damage, Impaired FANCL Expression, and Dysfunction in Pulmonary Vascular Endothelial Cells

Alkylating agents induce DNA crosslinks, and insufficient repair of these lesions leads to DNA damage accumulation, resulting in functional and phenotypic alterations in cells. In lung tissue from rats exposed to MMC (Figure 3A–D) or CDDP (Figure 3E–H) for 2 or 4 weeks, we observed downregulation of the core FA pathway factors FANCL and FANCD2, accompanied by upregulation of the DNA damage marker γ‐H2AX. As key components of the FA pathway, FANCL and FANCD2 are known to facilitate effective repair of alkylating agent‑induced DNA interstrand crosslinks (ICLs) and double‑strand breaks (DSBs) [24, 25, 26]. The expression of FANCL was also significantly downregulated in the lung tissue of classic MCT‐PAH rat model [17], suggestively an alkylating agent‐induced DNA crosslinking related model [17, 27], but only mildly decreased in classic PAH model induced by Sugen/Hypoxia (Figure S3). Subsequently, the downregulation of FANCL and FANCD2 in the pulmonary vascular endothelium was verified by IHC staining in lung tissues from rats treated with MMC or CDDP for 2 or 4 weeks (Figure 3I,J). Elevated γ‐H2AX levels in the pulmonary vascular endothelium were also observed in MMC/CDDP‑treated rats by IHC (Figure 3I,J) and IF (Figure 3K,L). In addition, IHC staining revealed increased FANCL/FANCD2 and γ‐H2AX in RV of MMC/CDDP‑treated rats, whereas the pro‑EndoMT transcription factor Twist showed a less pronounced increase (Figure S4A). TUNEL staining further demonstrated significantly increased apoptosis in the RV of these animals (Figure S4B). Collectively, these data suggest that MMC and CDDP differentially regulate DNA damage, apoptosis, and EndoMT in the pulmonary vasculature versus the right ventricle.

**FIGURE 3:**
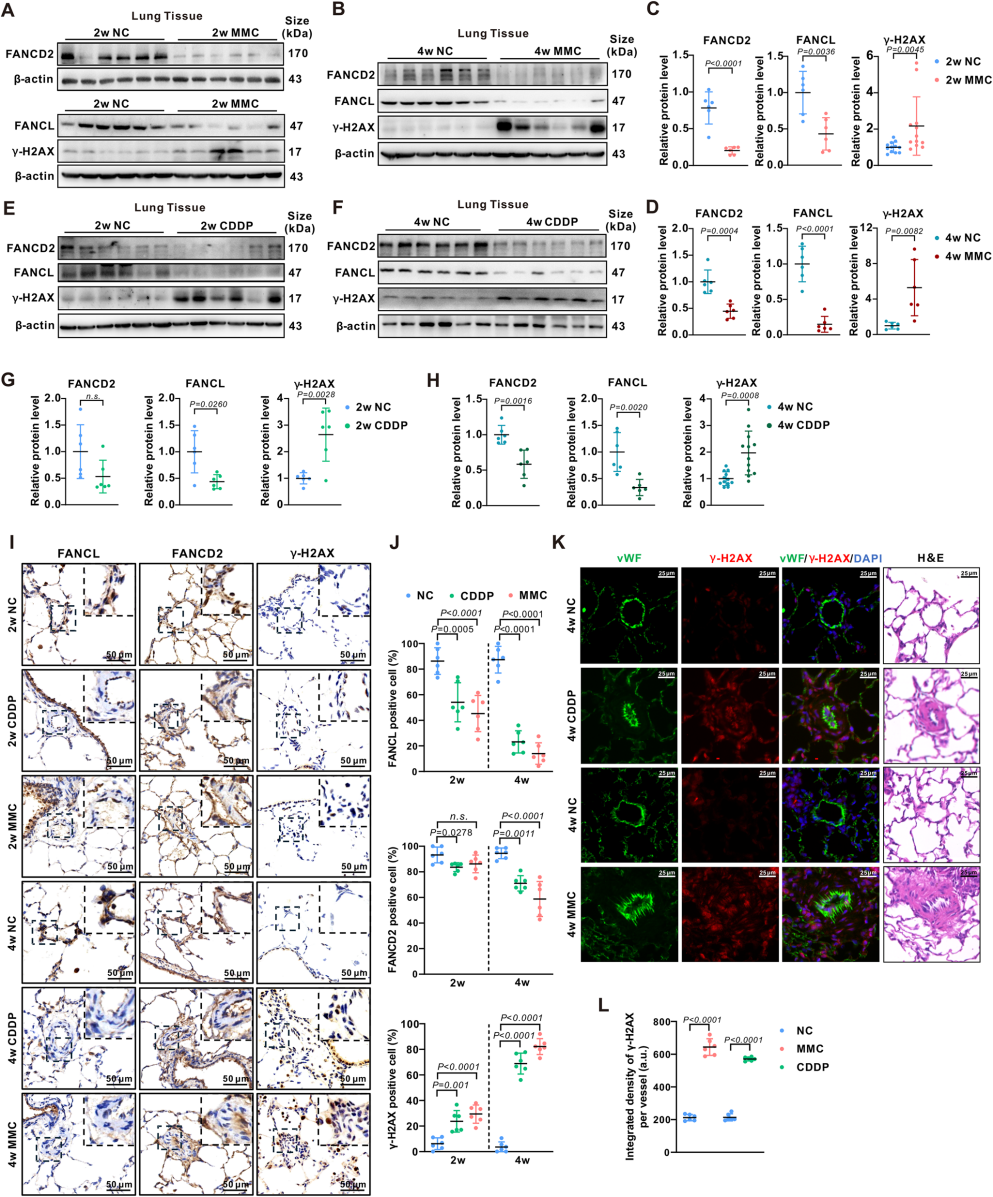
MMC or CDDP Treatment Induces Progressive DNA Damage Accumulation in Pulmonary Vascular Endothelium. (A–H) Immunoblots and analyzed graphs showing the γ‐H2AX protein expression in the lung tissue of rats treated with MMC (3 mg/kg, once weekly) for 2 or 4 weeks (A–D), and CDDP (4 mg/kg, once weekly) for 2 or 4 weeks (E–H). *n* = 6–12 rats per group. Protein expression levels were calculated as the ratio of the target protein band intensity to that of β‑actin and were normalized to the mean values of the respective 2‑week or 4‑week NC groups, expressed as fold change. (I, J) Immunohistochemical staining for the FA core factors (FANCL and FANCD2) and the DNA damage marker γ‐H2AX in lung sections from rats treated with MMC (3 mg/kg, once weekly) or CDDP (4 mg/kg, once weekly) for 2 or 4 weeks, followed by quantification of the percentage of positive cells within the pulmonary vasculature. *n* = 6 rats per group. (K, L) Immunofluorescence staining for the endothelial cell marker vWF and the DNA damage marker γ‐H2AX in lung sections from rats treated with MMC (3 mg/kg, once weekly) or CDDP (4 mg/kg, once weekly) for 4 weeks, followed by correlation analysis between γ‐H2AX fluorescence intensity within vWF‑positive regions and vessel wall area percentage. *n* = 6 rats per group, with 10–25 vessels analyzed per group. Data are presented as mean ± SD. Differences between two groups were analyzed by *t*‐test for C (FANCD2, FANCL), D (all panels), G (FANCD2, γ‐H2AX), H (all panels), and L (CDDP), Mann‐Whitney U‐test for C (γ‐H2AX), D (PCNA), G (FANCL), and L (MMC), or one‐way ANOVA with Tukey's honestly significant difference (Tukey's HSD) post hoc test for J. A value of *P* < 0.05 is considered statistically significant, n.s. indicates no statistical significance. Scale bar = 50 µm in I and 25 µm in K. NC: normal control.

At the cellular level, we further investigated the phenotypic and functional effects of MMC or CDDP exposure in freshly isolated PMVECs from each rat model group, using CD45^−^/CD31^+^ microbead‑based magnetic separation. Compared with PMVECs from control rats (NC‑PMVECs), those isolated from MMC‑ or CDDP‑treated rats (MMC‑PMVECs or CDDP‑PMVECs) displayed a morphological transition from the typical endothelial cobblestone appearance to a spindle‑shaped, fibroblast‑like phenotype, with a significantly increased aspect ratio after 2 or 4 weeks of exposure (Figure 4A,B). Concomitantly, a significantly higher proportion of these cells co‑expressed the mesenchymal marker α‑SMA and the endothelial marker CD31, indicating the occurrence of EndoMT (Figure 4C,D). Comet assay and flow cytometry analysis revealed markedly higher levels of baseline DNA damage (Figure 4E,F) and apoptosis (Figure 4G,H) in MMC‐PMVECs and CDDP‐PMVECs compared to NC‐PMVECs. MMC‐PMVECs also exhibited greater sensitivity to CDDP or MMC treatment at 8, 24, and 48 h (Figure S5). The upregulation of pro‐EndoMT markers Vimentin and Snail and DNA damage marker γ‐H2AX, downregulation of endothelial cell marker CD31 and GCN2 (encoded by *EIF2AK4*), a key regulator implicated in heritable PVOD [28], were validated by immunoblotting in MMC‐PMVECs and CDDP‐PMVECs, in contrast to NC‐PMVECs (Figure 4I,J).

**FIGURE 4:**
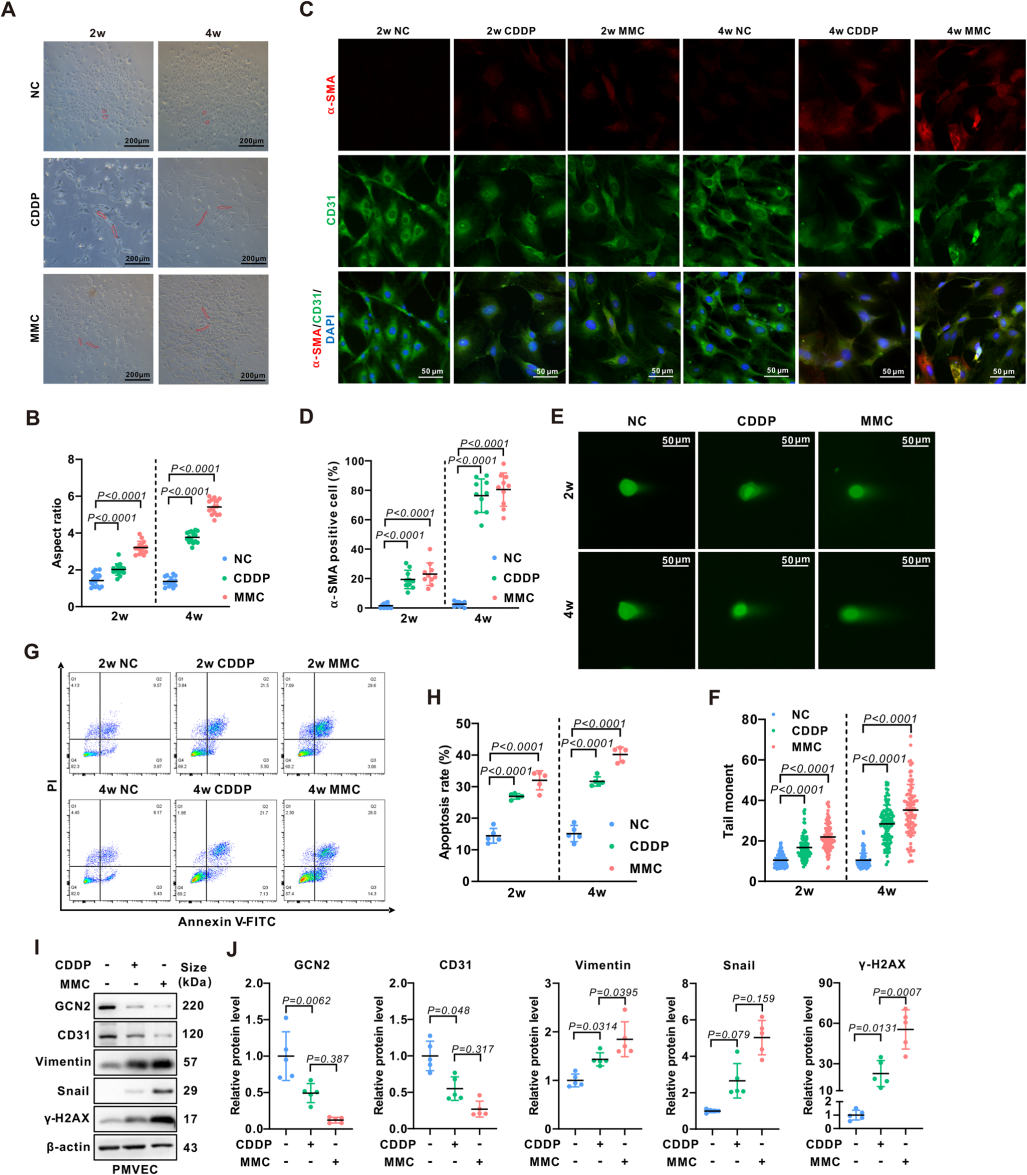
Enhanced EndoMT and Apoptosis in PMVECs Freshly Isolated from Rats Following MMC or CDDP Treatment. (A, B) Representative images and quantification of aspect ratio showing morphological changes in freshly isolated and primary cultured PMVECs at day 7, derived from rats treated with MMC (3 mg/kg, once weekly) or CDDP (4 mg/kg, once weekly) for 2 or 4 weeks. Data are based on 10–20 independent experiments per group. (C, D) Immunofluorescence staining for the endothelial marker CD31 and the mesenchymal marker α‑SMA, with quantification of the percentage of α‑SMA‑positive PMVECs isolated from rats following 2‐ or 4‑week MMC (3 mg/kg, once weekly) or CDDP (4 mg/kg, once weekly) treatment. *n* = 10 experiments per group. (E, F) Comet assay and quantification of tail moment showing DNA damage levels in freshly isolated PMVECs from rats treated with MMC (3 mg/kg, once weekly) or CDDP (4 mg/kg, once weekly) for 2 or 4 weeks. Data are based on *n* = 5 independent experiments, with approximately 100 cells analyzed per experiment per group. (G, H) Flow cytometric analysis and quantification of apoptosis in freshly isolated PMVECs from rats treated with MMC (3 mg/kg, once weekly) or CDDP (4 mg/kg, once weekly) for 2 or 4 weeks. *n* = 5 independent experiments per group. (I, J) Immunoblotting analysis of GCN2, CD31, Vimentin, Snail, and γ‐H2AX in PMVECs isolated from rats treated with MMC (3 mg/kg, once weekly) or CDDP (4 mg/kg, once weekly) for 4 weeks. *n* = 5 independent experiments per group. Data are presented as mean ± SD. Differences were analyzed by one‐way ANOVA with Tukey's honestly significant difference (Tukey's HSD) post hoc test for B (aspect ratio), D (α‑SMA positive cell), H (apoptosis rate), and L (GCN2, vimentin, γ‐H2AX), or Mann‐Whitney U‐test for F (tail moment) and L (CD31, Snail). A value of *P* < 0.05 is considered statistically significant, n.s. indicates no statistical significance. Scale bar = 200 µm in A, 50 µm in C, and 50 µm in E. NC: normal control.

To broaden the scope of our findings, we employed a customized dissection and culture strategy to successfully isolate and culture endothelial cells originating from distal pulmonary arteries (PAECs), pulmonary veins (PVECs), and lung microvessels (PMVECs) (Figure 5A–D). Upon exposure to MMC or CDDP, all three cell types exhibited convergent phenotypic changes, including elevated EndoMT evaluated by immunofluorescence staining for α‑SMA/CD31 (Figure 5E,F), apoptosis assessed by flow cytometry (Figure 5G,H), DNA damage by comet assay (Figure 5I,J), and impaired barrier junction by ZO‑1 immunofluorescence (Figure 6A,B), proliferation by Edu incorporation (Figure 6C,D) and angiogenic capacity by tube formation (Figure 6E,F). These findings were consistent with immunoblotting results for specific marker proteins associated with the above functional pathways (Figure 7A–L).

**FIGURE 5:**
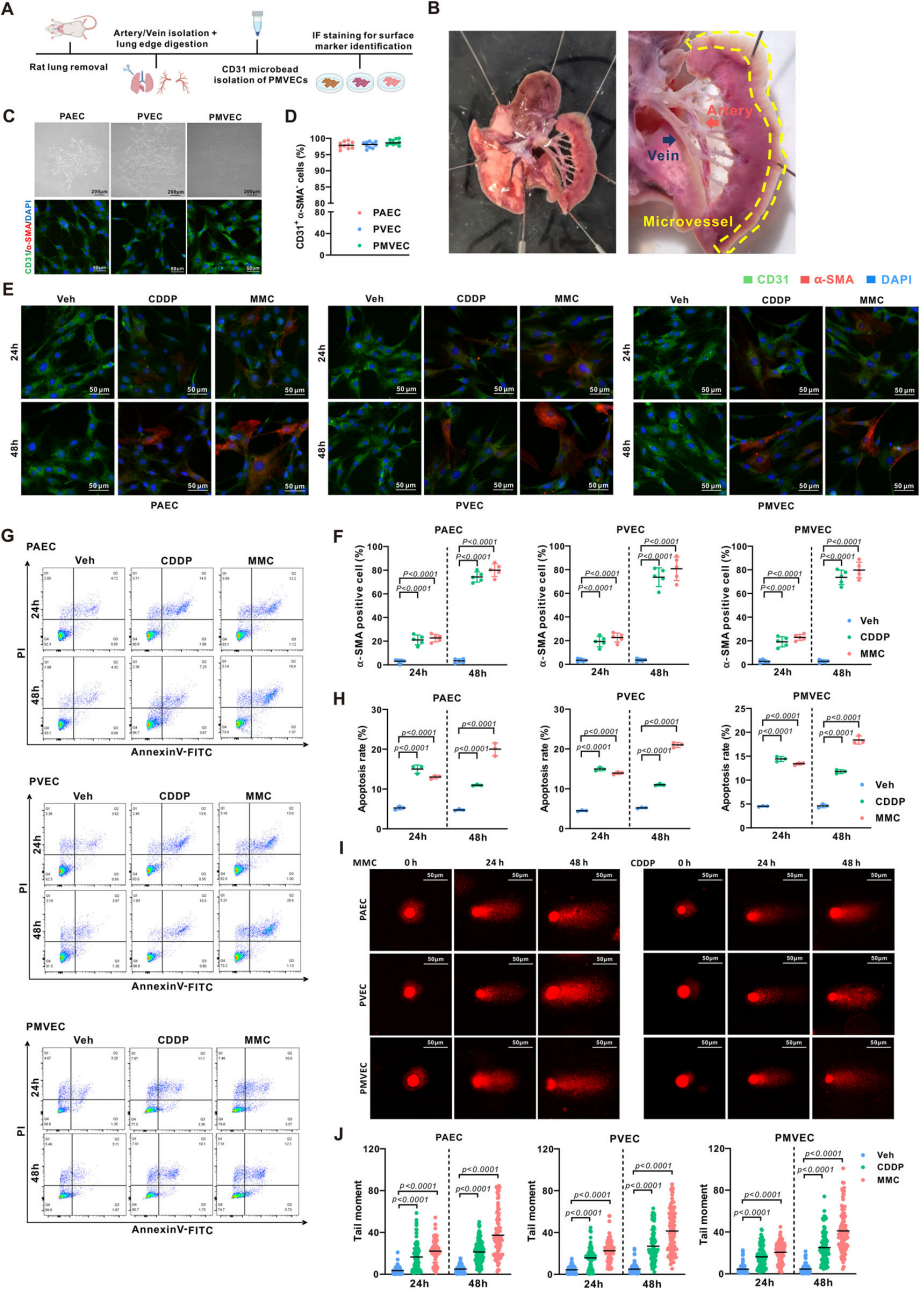
Effects of MMC and CDDP on EndoMT, Apoptosis, and DNA Damage in Primarily Cultured Rat PAECs, PVECs, and PMVECs. (A–D) Isolation, culture, and validation protocols for the primarily cultured PAECs, PVECs, and PMVECs. (E, F) Immunofluorescence staining for CD31 and α‑SMA, with quantification of the percentage of α‑SMA‑positive cells in PAECs, PVECs, and PMVECs treated with MMC (0.5 µg/mL) or CDDP (12 µM) for 24 or 48 h, *n* = 5 experiments per group. (G, H) Flow cytometric analysis and quantification of apoptosis in PAECs, PVECs and PMVECs treated with MMC (0.5 µg/mL) or CDDP (12 µM) for 24 or 48 h. *n* = 3 independent experiments per group. (I, J) Comet assay and quantification of tail moment showing DNA damage levels in PAECs, PVECs and PMVECs treated with MMC (0.5 µg/mL) or CDDP (12 µM) for 24 or 48 h. Data are based on *n* = 5 independent experiments, with approximately 100 cells analyzed per experiment per group. Data are presented as mean ± SD. Differences were analyzed by one‐way ANOVA with Tukey's honestly significant difference (Tukey's HSD) post hoc test for F (all panel) and H (PVEC, PMVEC), or Mann‐Whitney U‐test for H (PAEC), J (all panel). A value of *P* < 0.05 is considered statistically significant, n.s. indicates no statistical significance. Scale bar = 200 µm and 50 µm in C, 50 µm in E and I. Veh: vehicle control.

**FIGURE 6:**
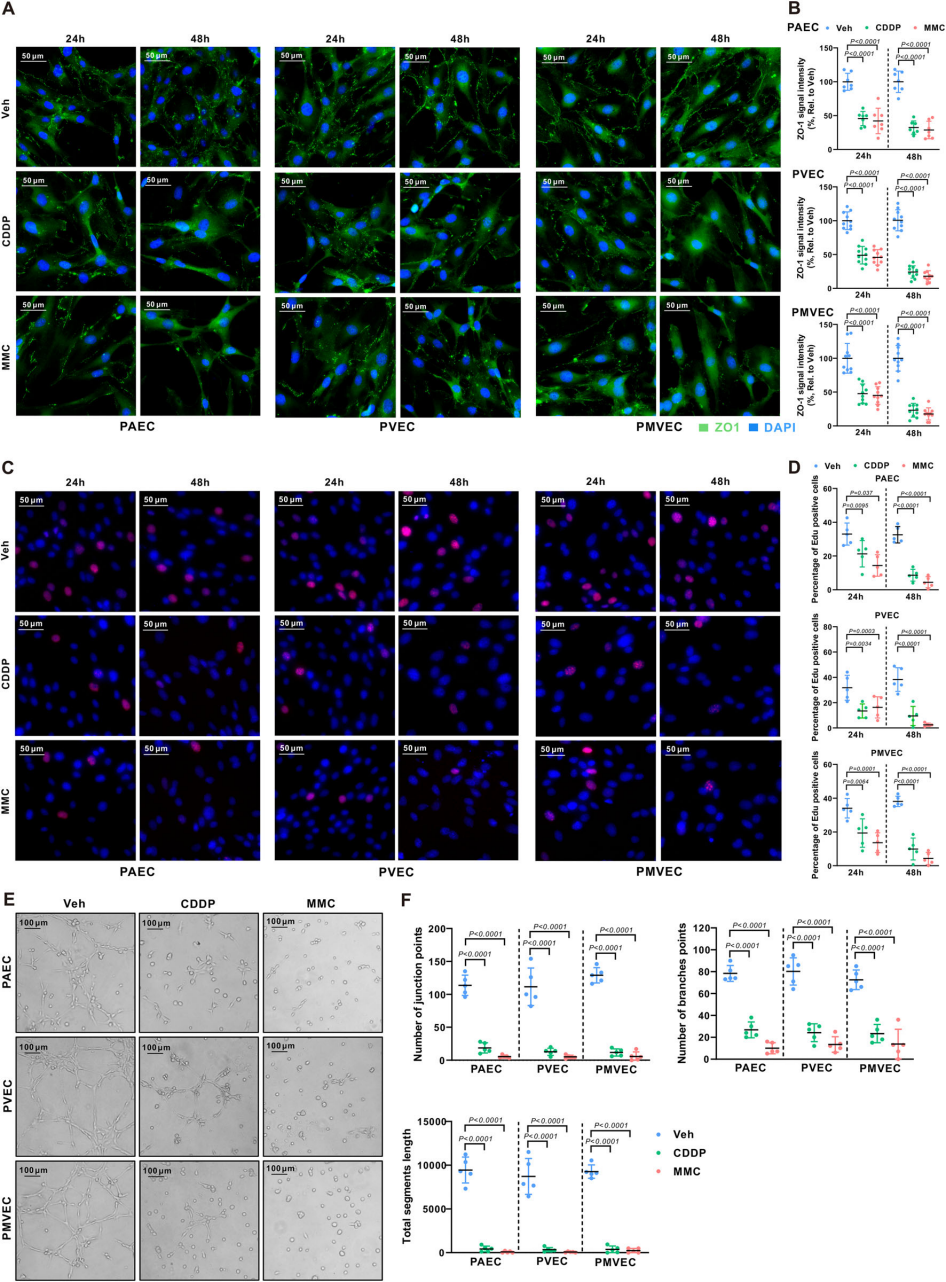
Effects of MMC and CDDP on Barrier Junction, Proliferation, and Tube Formation in Primarily Cultured Rat PAECs, PVECs, and PMVECs. (A, B) Immunofluorescence staining and quantification of ZO‑1 expression in PAECs, PVECs, and PMVECs treated with MMC (0.5 µg/mL) or CDDP (12 µM) for 24 or 48 h. *n* = 7 independent experiments for PAECs and *n* = 10 for PVECs and PMVECs. (C, D) Edu incorporation and quantification in PAECs, PVECs and PMVECs treated with MMC (0.5 µg/mL) or CDDP (12 µM) for 24 or 48 h. *n* = 5 independent experiments per group. (E, F) Tube formation images and quantification of the angiogenic capacity in PAECs, PVECs, and PMVECs treated with MMC (0.5 µg/mL) or CDDP (12 µM) for 24 or 48 h. *n* = 6 independent experiments per group. Data are presented as mean ± SD. Differences for B (ZO‐1 signal intensity) and D (EdU‐positive cell percentage) were analyzed by one‐way ANOVA with Tukey's HSD post hoc test. Differences between two groups in F were analyzed by unpaired *t*‐test, except for the comparison of total segment length between Veh‐ and MMC‐treated PMVECs, for which the Mann–Whitney U‐test was used. A value of *P* < 0.05 is considered statistically significant, n.s. indicates no statistical significance. Scale bar = 50 µm in A and C, and 100 µm in E. Veh: vehicle control.

**FIGURE 7:**
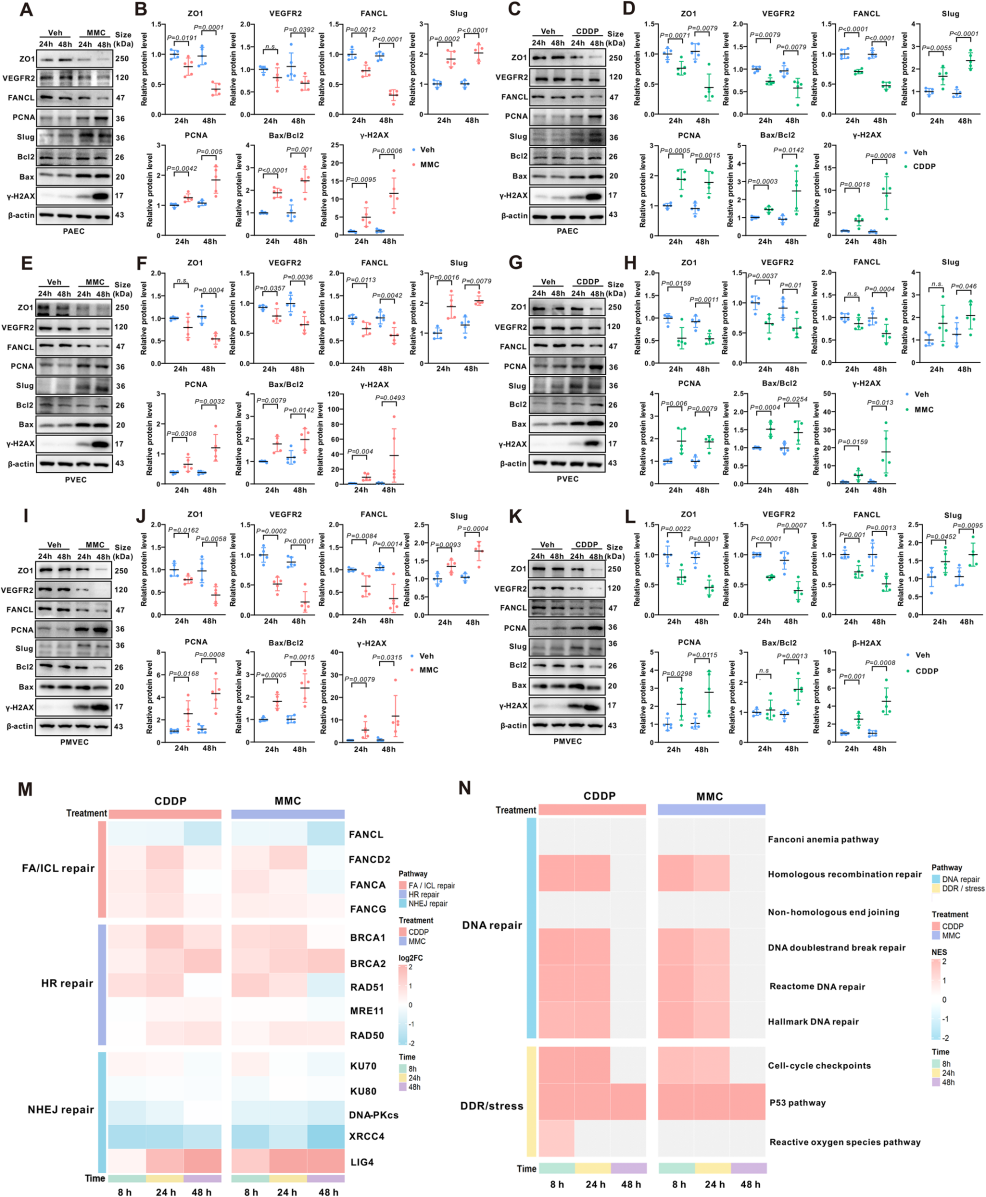
Effects of MMC and CDDP on Functional Marker Protein Expression in Primary Cultured Rat PAECs, PVECs, and PMVECs. (A–L) Immunoblots and analyzed graphs showing the protein expression of ZO‐1, VEGFR2, FANCL, PCNA, Slug, Bcl2, Bax, and γ‐H2AX in PAECs, PVECs, and PMVECs treated with MMC (0.5 µg/mL) or CDDP (12 µM) for 24 or 48 h. Data are presented as mean ± SD, *n* = 5 rats per group. Data are presented as mean ± SD. Differences between two groups were analyzed by unpaired *t*‐test, except for D (ZO1 at 48 h and VEGFR2 at 24 and 48 h), F (Bax/Bcl2 and γ‐H2AX at 24 h and Slug at 48 h), H (ZO1 at 24 h and PCNA at 48 h), and J (γ‐H2AX at 24 h), for which the Mann–Whitney U‐test was used. A value of *P* < 0.05 is considered statistically significant, n.s. indicates no statistical significance. (M, N) Heatmap of RNA‑seq data depicting gene‑ and pathway‑level changes in cultured rat PMVECs treated with MMC (0.5 µg/mL) or CDDP (12 µM) for 24 or 48 h. Veh: vehicle control.

Consistent with the findings in freshly isolated PMVECs and the three types of cultured lung endothelial cells, exposure to MMC or CDDP also reduced cell viability (Figure S6A,C) and increased apoptosis (Figure S6B,D) in primary cultured rat PMVECs, in both time‑ and dose‑dependent manners. Moreover, dose‑dependent upregulation of γ‐H2AX was observed following MMC or CDDP treatment (Figure S6E–H). Complementary assessment by γ‐H2AX immunofluorescence staining (Figure S6I,J) and comet assay (Figure S6K,L) further confirmed the progressive accumulation of MMC‑ or CDDP‑induced DNA damage. Similar effects on apoptosis, tube formation, and DNA damage were validated in cultured human PAECs (Figure S7A–J). In addition, immunohistochemical staining of lung sections revealed significantly elevated γ‐H2AX signals within the distal pulmonary vasculature of PVOD patients compared with control subjects (Figure S7K,L).

To further explore the transcriptional effects of MMC and CDDP, we performed RNA‐seq (Figure 7M,N) and PCR validation (Figure S8) to assess the expression of genes involved in three major DNA repair pathways: the non‐homologous end joining (NHEJ) pathway, the homologous recombination (HR) pathway, and the Fanconi anemia (FA) pathway. Comparative analysis revealed a conserved molecular signature, characterized by pronounced downregulation of FANCL in MMC/CDDP‐treated PMVECs (Figure 7M,N), which was further validated at protein level in PAECs, PVECs, and PMVECs following MMC or CDDP exposure (Figure 7A–L). As the primary mechanism for repairing alkylating agent‑induced DNA damage, impairment of the FA pathway leads to insufficient DNA repair, thereby exacerbating DNA damage accumulation and its downstream functional consequences. In primary cultured rat PMVECs, specific siRNA‑mediated knockdown of the FA core complex genes *Fancl* (Figure S9A,B) or *Fancd2* (Figure S9C,D) significantly increased apoptosis (Figure S9E,F) and DNA damage. This was evidenced by upregulated γ‐H2AX protein expression (immunoblotting; Figure S9A–D), increased γ‐H2AX foci (IF staining; Figure S9G,H), and elevated DNA fragmentation (comet assay; Figure S9I,J).

### 3.3 Mutual Regulation of FANCL and GCN2 in Primary Cultured Rat PMVECs

In MMC‑ or CDDP‑induced PAH rat models, we observed consistent downregulation of the FA core complex factors FANCL and FANCD2, along with the key PAH/PVOD regulator GCN2. To investigate their potential mutual regulation, primary cultured rat PMVECs were transfected with specific siRNA targeting FANCL (si*Fancl*) or GCN2 (si*Eif2ak4*). Knockdown of FANCL resulted in significant downregulation of GCN2 (Figure S10A,B), accompanied by increased apoptosis (Figure S10C,D) and elevated DNA damage (Figure S10A,B,E,F). Conversely, knockdown of GCN2 led to marked downregulation of FANCL (Figure S10G,H), together with increased apoptosis (Figure S10I,J) and DNA damage (Figure S10G,H,K,L). These findings reveal a previously unrecognized mutual regulation between GCN2 and FANCL and further implicate GCN2 in the modulation of DNA damage repair in PMVECs.

### 3.4 FANCL Rescue or Amifostine Treatment Attenuates Experimental PAH/PVOD in both Mice and Rats

Following the protocol outlined in Figure 8A, and consistent with our previous findings [17], FANCL deficiency in *Fancl* ^kf/kf^ mice not only drove spontaneous PAH/PVOD but also exacerbated CDDP‑induced disease in mice. AAV‑mediated FANCL rescue effectively ameliorated the PAH/PVOD phenotype in *Fancl* ^kf/kf^ mice exposed to CDDP, as evidenced by reduced RVSP (Figure 8B), improved Fulton index (Figure 8C), attenuated pulmonary vascular thickening (Figure 8D,E), and preserved right ventricular function and hypertrophy on echocardiography (Figure S11A–C).

**FIGURE 8:**
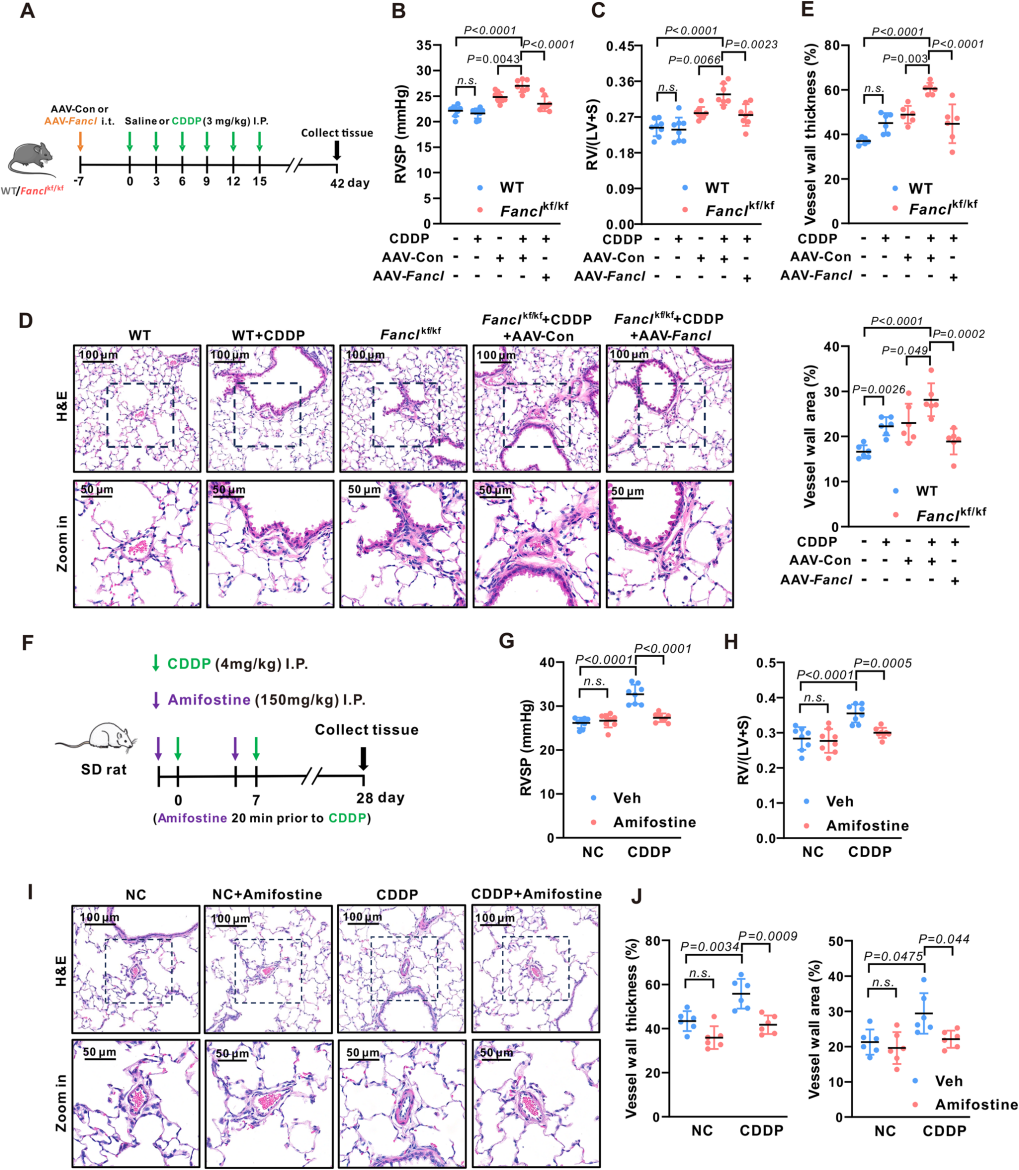
FANCL Rescue or Amifostine Treatment Attenuates Experimental PAH/PVOD in Both Mice and Rats. (A) Experimental timeline. (B, C) Quantification of RVSP (B) and Fulton index (C) in each mouse group. *n* = 7 mice per group. (D, E) Representative H&E staining with quantification of vessel wall thickness and vessel wall area in each group. *n* = 6 mice per group, with 20–30 vessels analyzed per group. (F) Experimental timeline. (G, H) Quantification of RVSP (G) and Fulton index (H), *n* = 8 rats in each group. (I, J) Representative H&E staining with quantification of vessel wall thickness and vessel wall area, *n* = 6 rats in each group, with 20–30 vessels analyzed per group. Data are mean ± SD. Significance assessed by one‐way ANOVA with Tukey's honestly significant difference (Tukey's HSD) post hoc test for B, C, and E (all panel), or two‐way ANOVA followed by a Tukey's HSD post hoc test for G, H, and J with an interaction *P* < 0.0001 (RVSP), *P* = 0.0201 (Fulton index), *P* = 0.1415 (vessel wall thickness), and *P* = 0.1215 (vessel wall area). A value of *P* < 0.05 is considered statistically significant, n.s. indicates no statistical significance. Scale bar = 100 µm and 50 µm in D and I.

Moreover, following the protocol outlined in Figure 8F, treatment of a DNA protector, Amifostine [17, 29], significantly normalized the CDDP‑induced elevation of RVSP (Figure 8H) and Fulton index (Figure 8I), attenuated pulmonary vascular thickening (Figure 8J,K), and improved right ventricular function and hypertrophy, as assessed by both echocardiography and histological analysis (Figure S12A–C).

## 4 Discussion

The occurrence of PAH/PVOD in cancer patients or transplant recipients treated with alkylating chemotherapeutic agents suggests a causative link. In this study, we established and characterized rat models of PAH/PVOD using MMC or CDDP. Both agents induced progressive PAH and pulmonary microvascular remodeling, although CDDP elicited milder pathology than MMC. Mechanistically, MMC and CDDP caused endothelial cell injury, phenotypic transition, excessive apoptosis, and impaired angiogenic capacity and proliferation in pulmonary arterial, venous, and microvascular endothelial cells, accompanied by conserved FANCL downregulation. Importantly, knockdown of either FANCD2 or FANCL led to elevated DNA damage and apoptosis. Notably, GCN2 knockdown increased DNA damage and apoptosis along with FANCL downregulation, revealing a previously unrecognized role for GCN2 in regulating DNA repair networks beyond its established function in PAH/PVOD. In vivo, *Fancl* deficiency in mice drove spontaneous and exacerbated CDDP‑induced disease, whereas AAV‑mediated FANCL rescue or amifostine treatment significantly attenuated PAH/PVOD in both mouse and rat models. Collectively, this study establishes a novel CDDP‑induced PAH/PVOD model, defines a conserved FANCL–DNA damage–endothelial dysfunction axis, and identifies FANCL rescue and DNA damage inhibition as potential therapeutic strategies for this severe PAH subtype.

Alkylating agents are highly reactive compounds that form covalent bonds with electron‐rich sites in biological molecules. They readily bind to DNA strands, forming crosslinks that impair normal DNA replication and RNA transcription. Alkylating agents are widely used as conventional chemotherapeutic agents to treat various malignancies, including lymphoma, Hodgkin's disease, breast cancer, and multiple myeloma, and are also utilized in transplant conditioning regimens [30]. The occurrence of PAH/PVOD in cancer patients or transplant recipients treated with chemotherapy, particularly alkylating agents, strongly suggests a correlation between alkylating chemotherapeutic agents and PAH/PVOD development [13, 31, 32, 33, 34]. While PVOD is classified within Group 1 PAH, PAH and PVOD remain distinct disease entities with fundamentally different pathophysiological mechanisms, clinical features, and therapeutic responses. PAH primarily involves precapillary pulmonary arterioles with plexiform lesions, whereas PVOD is characterized by fibrous intimal proliferation of septal veins and preseptal venules, often accompanied by pulmonary capillary dilatation and proliferation (pulmonary capillary hemangiomatosis‐like changes). According to the latest PH classification according to the 7^th^ WSPH, PVOD is classified as a subgroup 1.5, PAH with features of venous/capillary (PVOD/PCH) involvement, and MMC has been reported to be associated with PAH with features of PVOD/PCH involvement [5].

Analysis of the French PH Network Registry revealed large part of the chemotherapy‐associated PAH/PVOD cases involved alkylating agent exposure, primarily CP, MMC, CDDP, and 5‐Fluorouracil [13, 35]. These agents induce characteristic PAH/PVOD pathology and long‐term clinical outcomes [35]. Building on this evidence, subsequent studies, including our own, have sought to establish experimental PAH/PVOD models using alkylating agent‐treated animals to investigate underlying mechanisms. Perros et al. and our prior works similarly demonstrated MMC‐induced PAH/PVOD in rats [14, 15, 17] and mice [17]. A comparative study further revealed that the MMC‐induced rat model recapitulates key histological and molecular features of human PVOD [16]. Among the 37 chemotherapy‐associated PVOD cases, CDDP exposure was identified in 21.6% of patients—comparable to MMC (24.3%)—making it the third most frequently implicated alkylating agent. This clinical association establishes CDDP as a relevant PVOD trigger beyond MMC. Here, we establish for the first time that this CDDP model recapitulates the hallmark features of PVOD, including right ventricular hypertrophy, distal pulmonary vascular remodeling, venous involvement, and capillary proliferation—phenotypes strikingly similar to those of the established MMC model. This demonstrates that CDDP can reproduce the full spectrum of PVOD pathology in vivo, providing a complementary tool to study chemotherapy‐induced PVOD. This model enables comparative mechanistic studies between two distinct alkylating agents, helps dissect agent‐specific versus shared PVOD pathways, and expands the preclinical platform for testing therapeutic interventions across different PVOD‐inducing insults.

As classical chemotherapeutic agents, alkylating compounds generate substantial DNA ICLs and DSBs in both malignant and healthy cells. In mammalian systems, the FA pathway serves as the primary mechanism for repairing alkylating agent‐induced DNA damage. FA represents a multisystem genetic disorder characterized by congenital abnormalities, impaired repair of DNA ICLs/DSBs, and cancer predisposition [24]. To date, mutations in over twenty genes are known to cause FA, and their encoded proteins collectively constitute the FA pathway [24]. Cells deficient in this pathway exhibit heightened susceptibility to DNA ICLs induced by endogenous or exogenous sources—including alkylating agents [36]. During DNA replication, ICLs recruit and activate the FA core complex. This triggers monoubiquitination of the FANCI/FANCD2 heterodimer by the E3 ubiquitin ligase FANCL [24]. Activated FA signaling subsequently mobilizes classical DNA repair pathways—HR, NHEJ, and alternative end‐joining (Alt‐EJ)—to orchestrate ICL and DSB repair [24, 37]. Notably, our prior work demonstrated that *Fancl* knockout induces spontaneous PAH and heightens susceptibility to MMC‐induced PAH, characterized by severe DNA damage and apoptosis in PMVECs [17]. Mechanistically, *Fancl* deficiency suppresses bone morphogenetic protein (BMP) signaling while hyperactivating transforming growth factor beta (TGF‐β) pathways, constituting a well‐established pathogenic mechanism driving PAH development [17]. In this study, RNA‑seq, RT‑qPCR, and immunoblotting consistently demonstrated that MMC/CDDP treatment profoundly downregulated FANCL—a core component of the FA DNA repair pathway—in PAECs, PVECs and PMVECs. In vivo FANCL deficiency confirmed that impairment of this pathway exacerbates endothelial cell damage and promotes PAH/PVOD pathogenesis, whereas FANCL restoration or DNA damage protection effectively attenuates these pathological processes. FA pathway deficiency and/or MMC treatment induces sustained production and secretion of TGF‑β1. This paracrine TGF‑β1 not only further induces γ‑H2AX expression but also exacerbates PMVEC apoptosis and PASMC proliferation in the context of FANCL deficiency and/or MMC exposure, as demonstrated in our previous study [17], suggesting the existence of a positive feedback loop. This observation aligns with our earlier findings that MMC treatment significantly upregulates TGF‑β1 production and secretion, promoting PMVEC EndoMT and contributing to PAH/PVOD development [15]. Collectively, these data provide a mechanistic explanation for the transition from acute DNA damage to a sustained pro‑PAH/PVOD phenotype.

Pathogenic mutations are identified in approximately 13.3% of PVOD/PCH patients, with *EIF2AK4* (encoding GCN2) being the most frequently mutated gene [38]. GCN2 is a kinase integral to the integrated stress response. It senses various stresses and phosphorylates eIF2α, thereby regulating the expression of genes involved in adaptation, survival, and apoptosis across multiple cell types, including endothelial cells [16, 39, 40, 41, 42]. Although the association between biallelic *EIF2AK4* mutations and PAH/PVOD has been recognized for nearly two decades [28], the precise role of *EIF2AK4* (GCN2) in the pulmonary vasculature remains largely undefined. Previous studies demonstrated GCN2 downregulation in lung tissues from patients with heritable or sporadic PVOD, heritable or idiopathic PAH [43], as well as in MMC‐induced PAH/PVOD rat models [14, 15, 16]. However, the role of GCN2 in PAH/PVOD remains controversial. Despite the downregulation of GCN2 in PAH/PVOD, Manaud et al. reported that GCN2‐knockout rats did not develop PAH/PVOD but exhibited dramatic suppression of BMP‐Smad1/5/9 pathway [16]. Zhu et al. reported the GCN2 kinase activation triggers pulmonary vascular remodeling and PAH and that genetic disruption or pharmacologically inhibition of GCN2 kinase activity induced neither PVOD nor PAH but improved the hypoxia/MCT‐induced PAH models [44]. In a recent study, Zhang et al. reported a novel mechanism that GCN2 deficiency can induce macrophage ferroptosis and mediates the pulmonary venous arterialization [45]. The results of this study provide novel and essential evidence indicating that GCN2 participates in and plays an important role in regulating the cellular DNA damage repair mechanism in PMVECs. This regulation occurs, at least partially, through modulation of FANCL. Given that deficient FANCL induces suppressed BMPR2 pathway and activated TGF‐β pathway [17], and that loss‐of‐function mutations in BMPR2 exhibits increased susceptibility to MMC‐induced PVOD [46], the sustained suppression of FANCL, DNA damage accumulation and imbalance of BMPR2/TGF‐β pathways may represent a novel mechanism through which GCN2 contributes to PAH/PVOD pathogenesis.

Although this study provides important translational insights by demonstrating that established MMC‑ or CDDP‑induced PAH/PVOD rat models share substantial features with clinical observations in PAH/PVOD patients, these models also exhibit significant remodeling across all pulmonary microvessel types, including veins, arteries, and capillaries. This finding aligns with data from other group [46] and our prior observations in the MMC‐induced PAH mouse model [17]. This broad effect may reduce the specificity of these models for mechanistic studies focused on identifying PVOD‐specific pathophysiological venous and capillary signatures, and translational studies aimed at developing PVOD‐targeted therapeutics. Discrepancies between animal models and human diseases are a well‐recognized challenge. Consequently, developing more advanced, human‐relevant models remains a critical objective in preclinical research, requiring continuous effort and warranting further collaboration [47].

As summarized in the **Graphic Abstract**, these data not only provide a systematic evaluation of two clinically relevant alkylating agent‑induced PAH/PVOD rat models, but also strongly support a mechanistic cascade linking alkylating agent‑induced DNA damage accumulation, sustained FANCL suppression and consequent impairment of the FA DNA repair machinery, persistent endothelial cell injury, and progressive pulmonary microvascular remodeling leading to PAH/PVOD pathogenesis. This framework, originally suggested by clinical observations, substantially advances our understanding of alkylating agent‑induced PAH/PVOD.

## Author Contributions

S.L., Y.S., X.W., and H.C. contributed to the data curation, formal analysis, investigation, methodology, software, validation, visualization; S.M., Z.L., Y.G., X.S., Y.L., Y.G., J.Z., H.Y., M.D., and F.W. contributed to the investigation, methodology, software; P.O., Z.W., C.Z., L.X., and W.L. contributed to the methodology, resources, software, project administration; J.W. contributed to the conceptualization, funding acquisition, project administration; K.Y. contributed to the conceptualization, funding acquisition, methodology, project administration, resources, supervision, Writing – original draft, Writing – review & editing; All authors approved for the submission of the manuscript.

## Conflicts of Interest

The authors declare no conflicts of interest.

## Supporting information




**Supporting File 1**: advs78081‐sup‐0001‐SuppMat.docx.

**Supporting File 2**: advs78081‐sup‐0002‐S1‐S12‐R1.pdf.

## Data Availability

The data that support the findings of this study are available from the corresponding author upon reasonable request.
