# Supplementary material for: Alkylating Agent–Induced FANCL Impairment Damages Pulmonary Arterial, Venous, and Microvascular Endothelial Cells and Predisposes to PAH/PVOD
Source: Adv Sci (Weinh). 2026 Oct 8:e78081. Online ahead of print. doi: 10.1002/advs.78081 (PMC13650023; doi:10.1002/advs.78081)
Supplement: Supplementary file 1 [file ADVS-9999-e78081-s002.pdf]

Figure S1

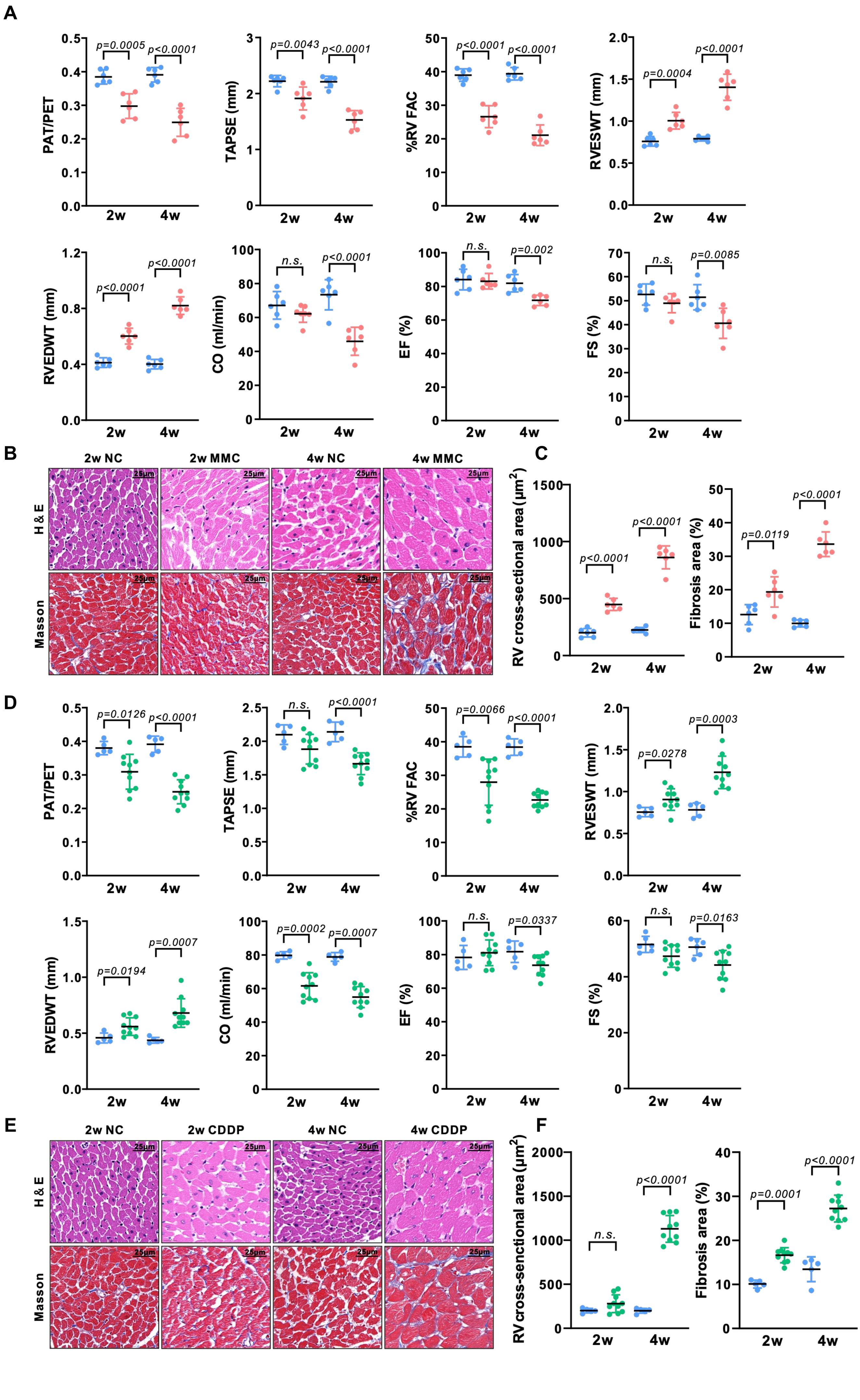
A
PAT/PET
TAPSE (mm)
%RV FAC
RVESWT (mm)
RVEDWT (mm)
CO (ml/min)
EF (%)
FS (%)
2w
4w
p=0.0005
p<0.0001
p=0.0043
p=0.0004
p=0.002
p=0.0085
n.s.
B
2w NC
2w MMC
4w NC
4w MMC
H & E
Masson
25μm
C
RV cross-sectional area (μm²)
Fibrosis area (%)
p=0.0119
D
p=0.0126
p=0.0066
p=0.0003
p=0.0278
p=0.0007
p=0.0194
p=0.0002
p=0.0337
p=0.0163
E
2w CDDP
4w CDDP
F
p=0.0001

Figure S2

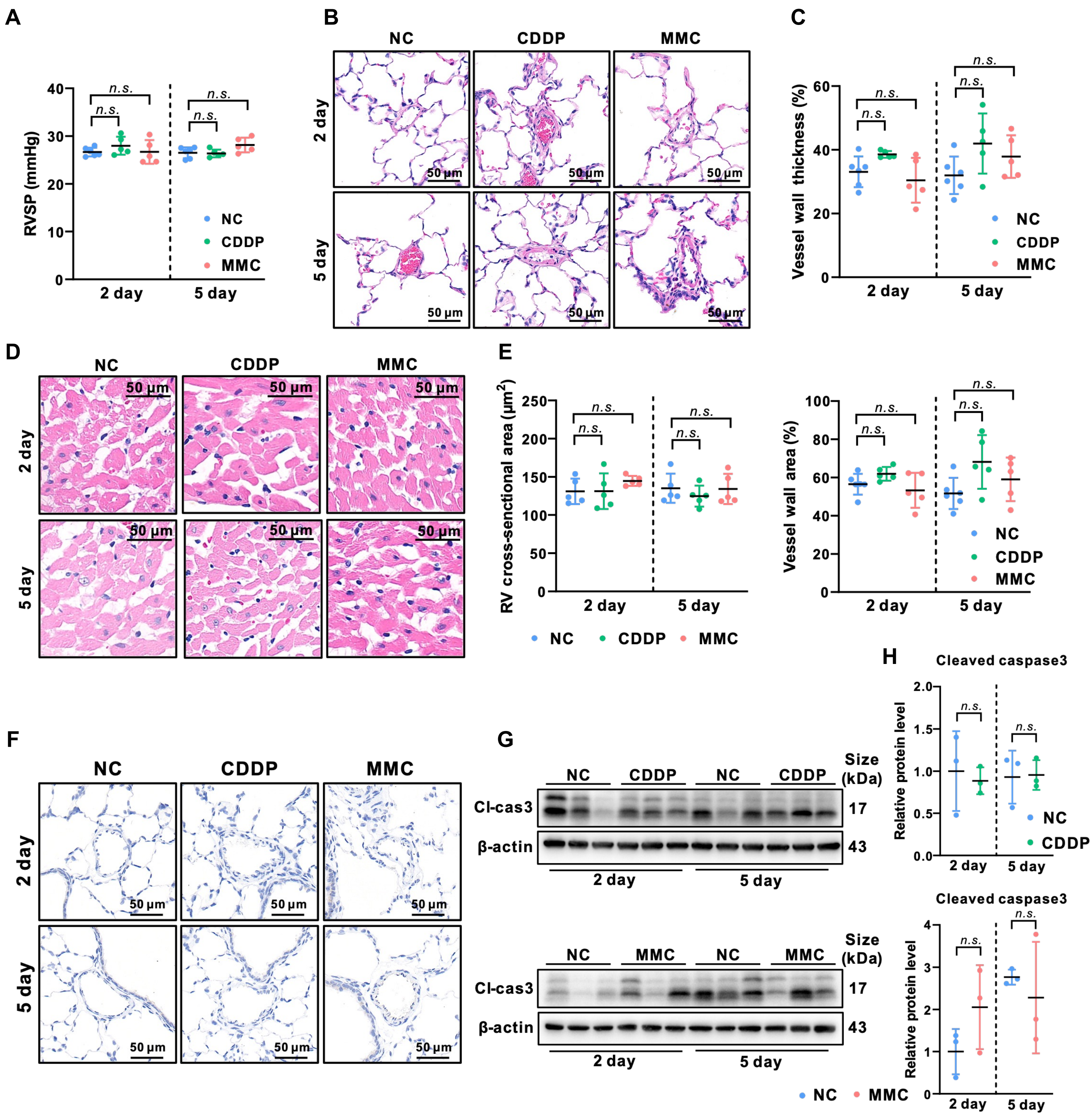

A
RVSP (mmHg)
n.s.
2 day
5 day
NC
CDDP
MMC
B
NC
CDDP
MMC
2 day
5 day
50 μm
C
Vessel wall thickness (%)
D
E
RV cross-sentional area (μm²)
Vessel wall area (%)
F
G
Size (kDa)
Cl-cas3
β-actin
17
43
H
Cleaved caspase3
Relative protein level

Figure S3

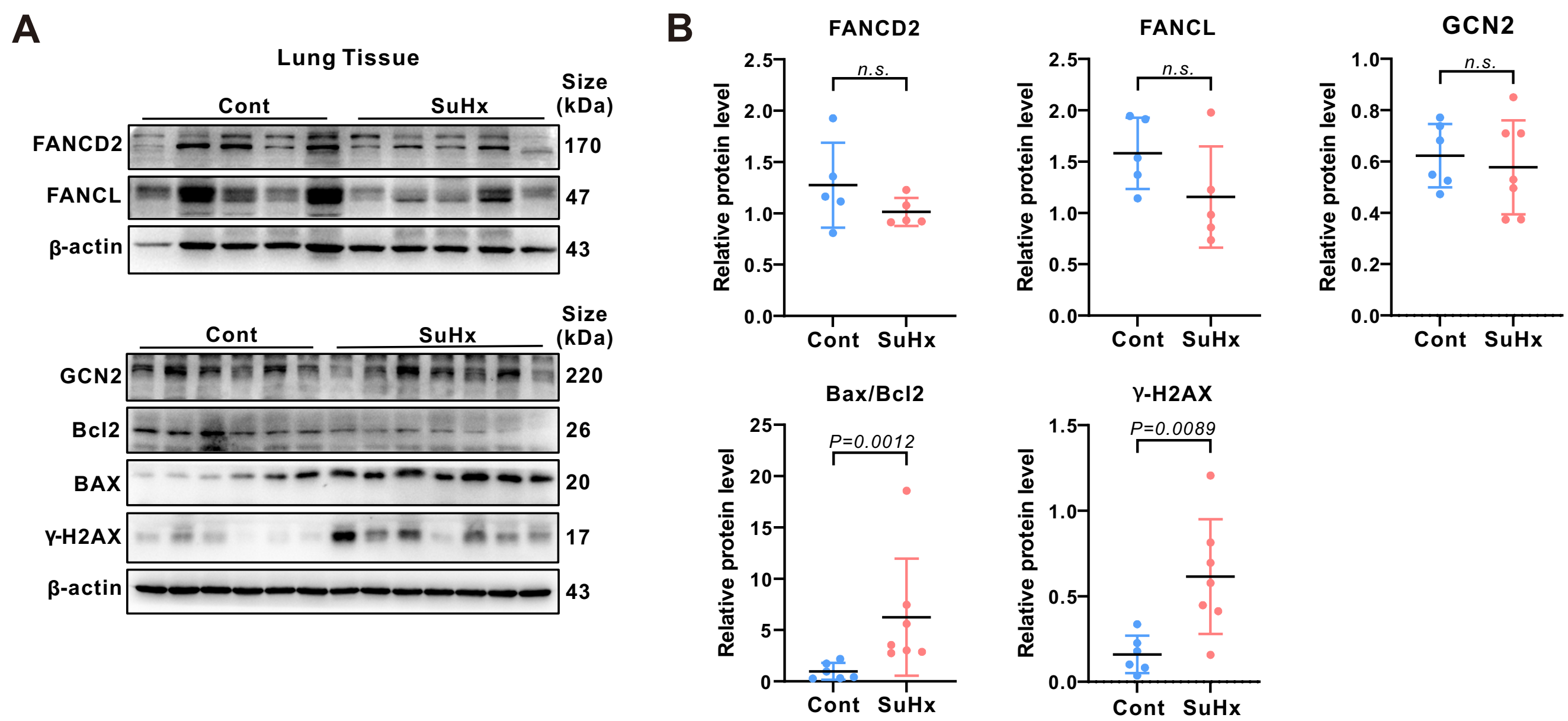
A
Lung Tissue
Cont
SuHx
Size (kDa)
FANCD2
170
FANCL
47
β-actin
43
GCN2
220
Bcl2
26
BAX
20
γ-H2AX
17
β-actin
43
B
FANCD2
FANCL
GCN2
Bax/Bcl2
γ-H2AX
Relative protein level
n.s.
P=0.0012
P=0.0089
Cont
SuHx

Figure S4

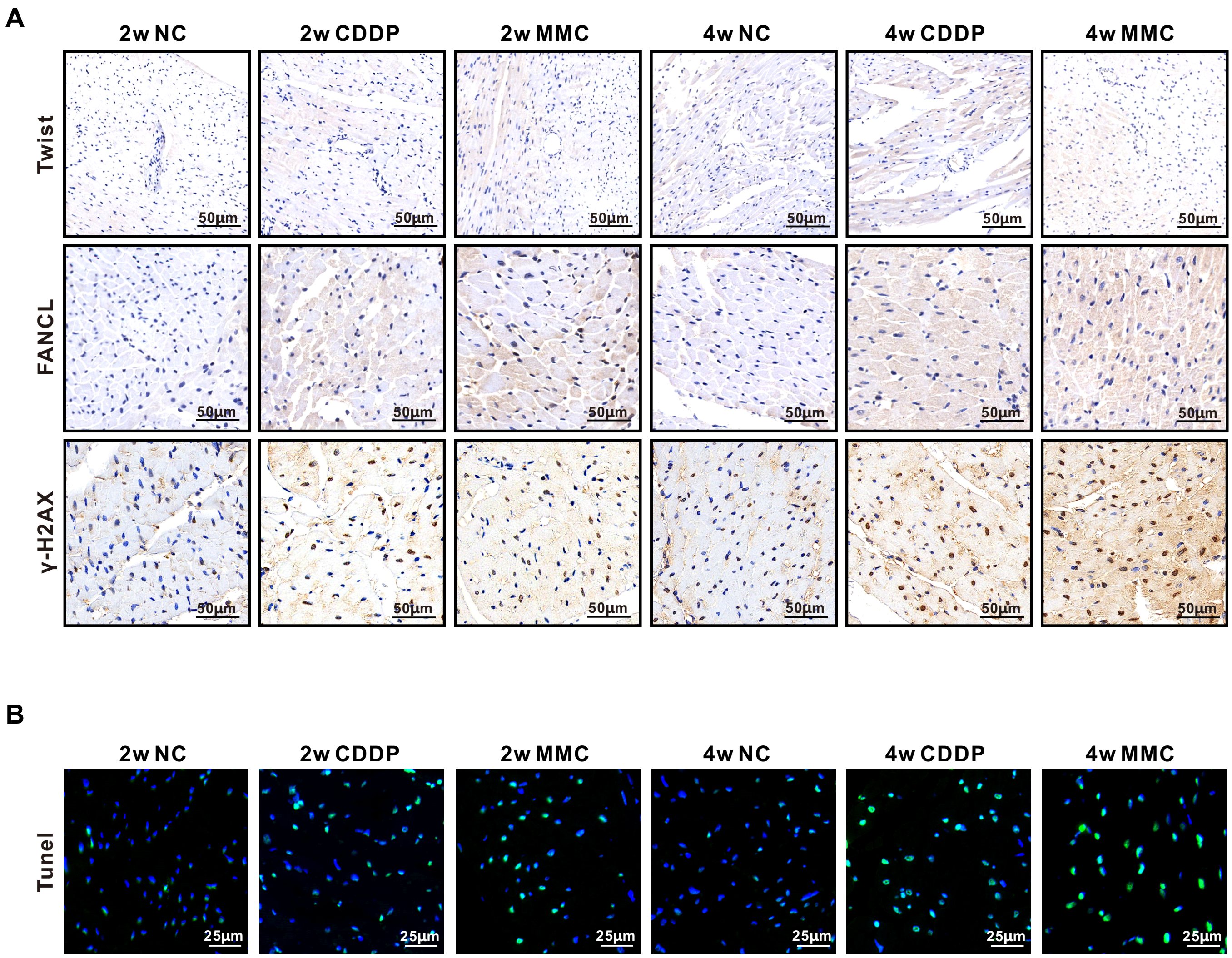
A
2w NC
2w CDDP
2w MMC
4w NC
4w CDDP
4w MMC
Twist
FANCL
γ-H2AX
50μm
B
2w NC
2w CDDP
2w MMC
4w NC
4w CDDP
4w MMC
Tunel
25μm

Figure S5

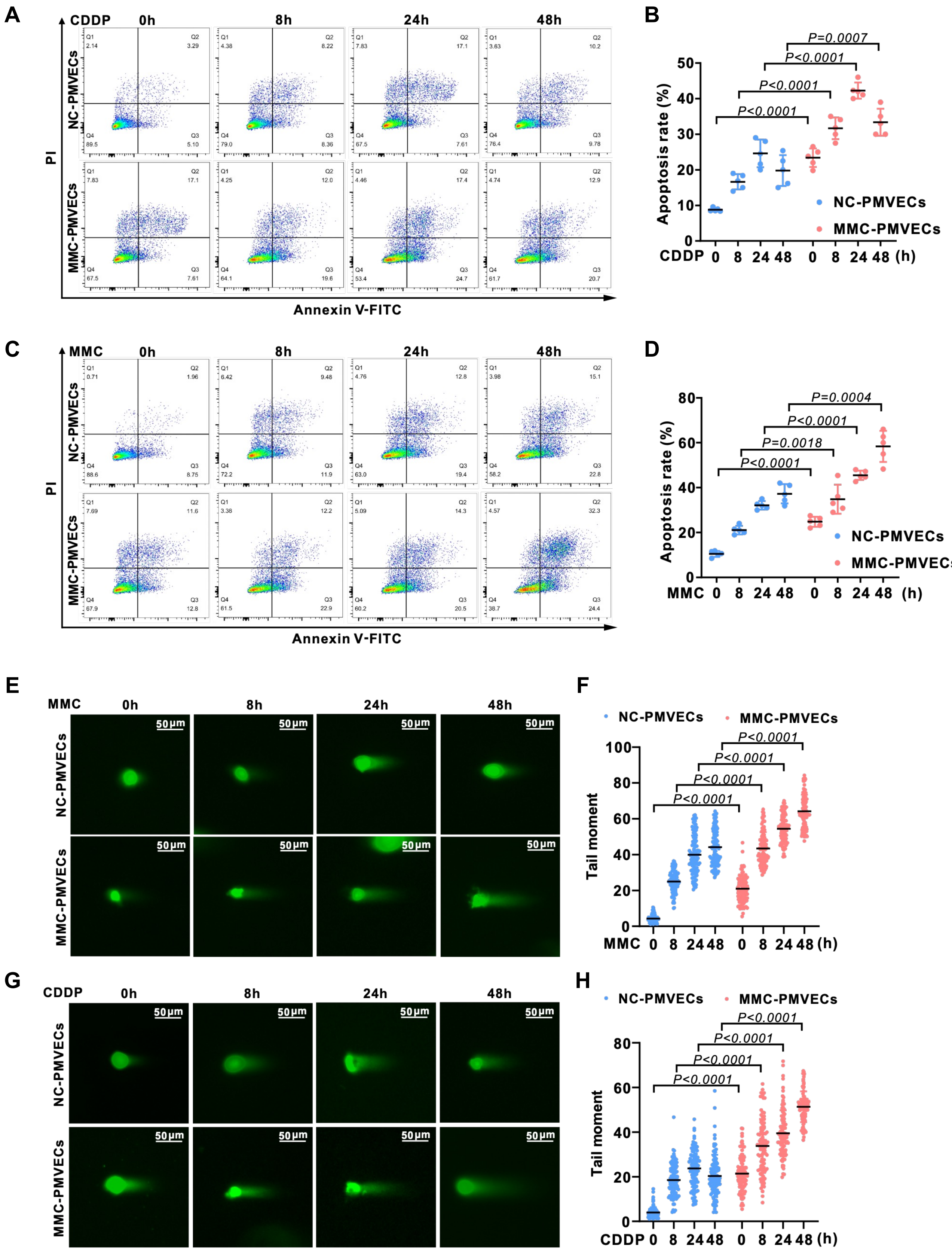
A
CDDP
0h
8h
24h
48h
NC-PMVECs
MMC-PMVECs
PI
Annexin V-FITC
B
Apoptosis rate (%)
P=0.0007
P<0.0001
P<0.0001
P<0.0001
NC-PMVECs
MMC-PMVECs
CDDP 0 8 24 48 0 8 24 48 (h)
C
MMC
0h
8h
24h
48h
NC-PMVECs
MMC-PMVECs
PI
Annexin V-FITC
D
Apoptosis rate (%)
P=0.0004
P<0.0001
P=0.0018
P<0.0001
NC-PMVECs
MMC-PMVECs
MMC 0 8 24 48 0 8 24 48 (h)
E
MMC
0h
8h
24h
48h
NC-PMVECs
MMC-PMVECs
50μm
F
NC-PMVECs
MMC-PMVECs
Tail moment
P<0.0001
P<0.0001
P<0.0001
P<0.0001
MMC 0 8 24 48 0 8 24 48 (h)
G
CDDP
0h
8h
24h
48h
NC-PMVECs
MMC-PMVECs
50μm
H
NC-PMVECs
MMC-PMVECs
Tail moment
P<0.0001
P<0.0001
P<0.0001
P<0.0001
CDDP 0 8 24 48 0 8 24 48 (h)

Figure S6

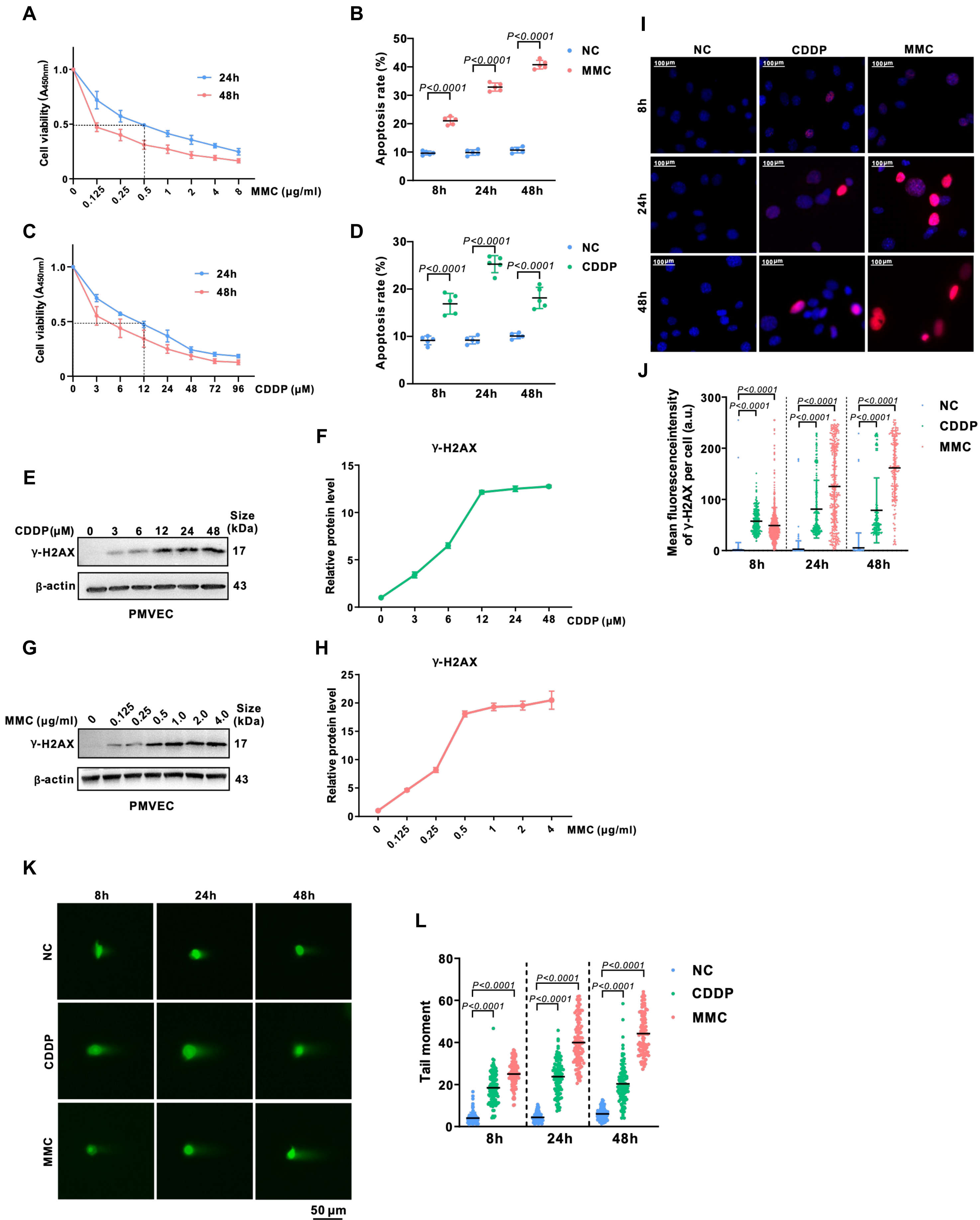
A
Cell viability (A450nm)
24h
48h
MMC (μg/ml)
0
0.125
0.25
0.5
1
2
4
8
B
Apoptosis rate (%)
P<0.0001
NC
MMC
8h
24h
48h
C
Cell viability (A450nm)
24h
48h
CDDP (μM)
0
3
6
12
24
48
72
96
D
Apoptosis rate (%)
P<0.0001
NC
CDDP
8h
24h
48h
E
CDDP(μM)
0
3
6
12
24
48
Size (kDa)
γ-H2AX
17
β-actin
43
PMVEC
F
γ-H2AX
Relative protein level
CDDP (μM)
G
MMC (μg/ml)
0
0.125
0.25
0.5
1.0
2.0
4.0
Size (kDa)
γ-H2AX
17
β-actin
43
PMVEC
H
γ-H2AX
Relative protein level
MMC (μg/ml)
I
NC
CDDP
MMC
8h
24h
48h
100μm
J
Mean fluorescenceintensity of γ-H2AX per cell (a.u.)
P<0.0001
NC
CDDP
MMC
8h
24h
48h
K
8h
24h
48h
NC
CDDP
MMC
50 μm
L
Tail moment
P<0.0001
NC
CDDP
MMC
8h
24h
48h

Figure S7

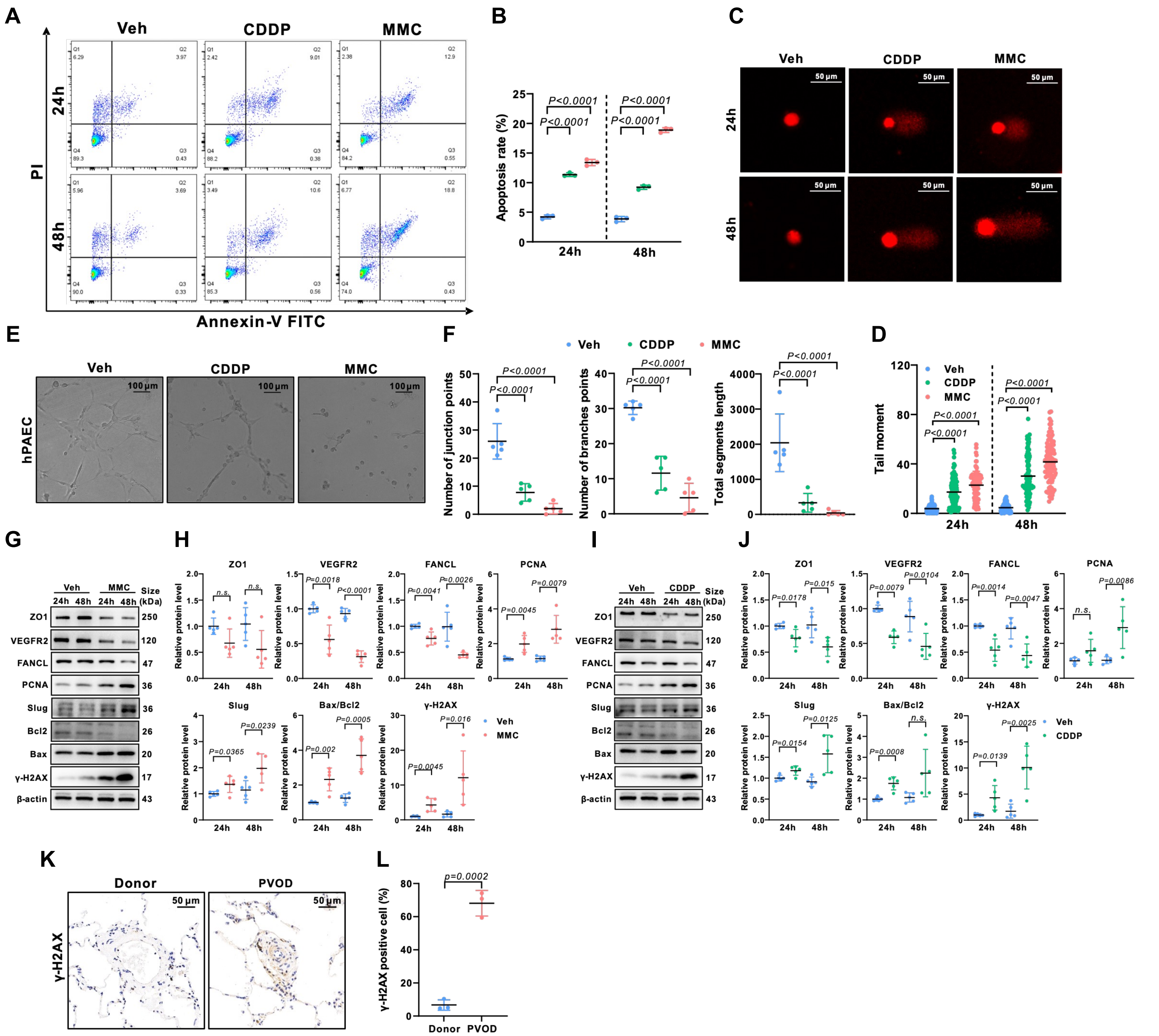
A
Veh
CDDP
MMC
24h
48h
PI
Annexin-V FITC
B
Apoptosis rate (%)
P<0.0001
24h
48h
C
Veh
CDDP
MMC
24h
48h
50 μm
D
Tail moment
Veh
CDDP
MMC
P<0.0001
24h
48h
E
Veh
CDDP
MMC
hPAEC
100 μm
F
Veh
CDDP
MMC
Number of junction points
Number of branches points
Total segments length
P<0.0001
G
Veh
MMC
24h 48h 24h 48h
Size (kDa)
ZO1 250
VEGFR2 120
FANCL 47
PCNA 36
Slug 36
Bcl2 26
Bax 20
γ-H2AX 17
β-actin 43
H
ZO1
VEGFR2
FANCL
PCNA
Slug
Bax/Bcl2
γ-H2AX
Relative protein level
n.s.
P=0.0018
P<0.0001
P=0.0041
P=0.0026
P=0.0045
P=0.0079
P=0.0365
P=0.0239
P=0.002
P=0.0005
P=0.0045
P=0.016
Veh
MMC
24h
48h
I
Veh
CDDP
24h 48h 24h 48h
Size (kDa)
ZO1 250
VEGFR2 120
FANCL 47
PCNA 36
Slug 36
Bcl2 26
Bax 20
γ-H2AX 17
β-actin 43
J
ZO1
VEGFR2
FANCL
PCNA
Slug
Bax/Bcl2
γ-H2AX
Relative protein level
P=0.0178
P=0.015
P=0.0079
P=0.0104
P=0.0014
P=0.0047
n.s.
P=0.0086
P=0.0154
P=0.0125
P=0.0008
n.s.
P=0.0139
P=0.0025
Veh
CDDP
24h
48h
K
Donor
PVOD
γ-H2AX
50 μm
L
γ-H2AX positive cell (%)
p=0.0002
Donor
PVOD

Figure S8

A

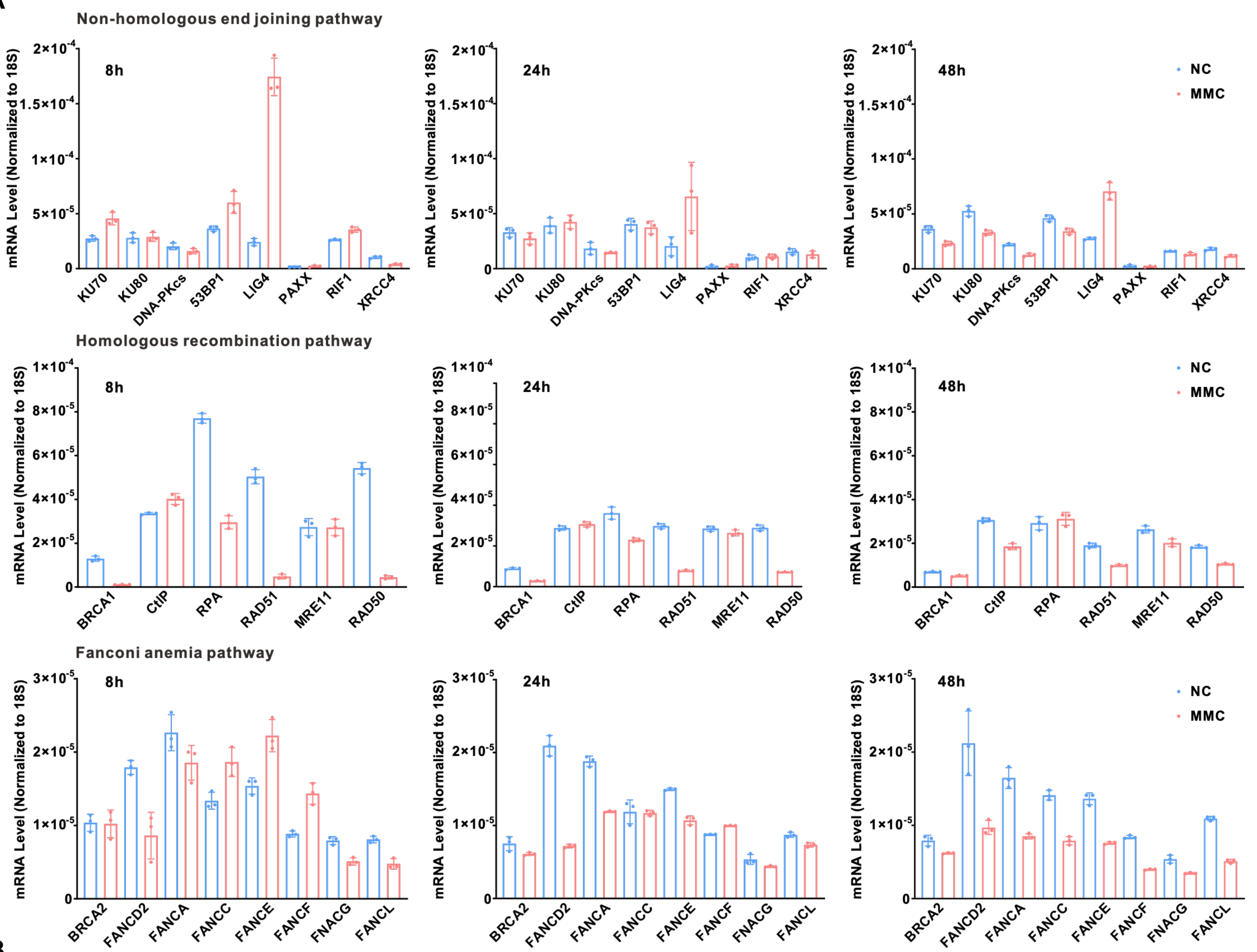

Non-homologous end joining pathway
8h
24h
48h
NC
MMC
mRNA Level (Normalized to 18S)
KU70
KU80
DNA-PKcs
53BP1
LIG4
PAXX
RIF1
XRCC4
Homologous recombination pathway
BRCA1
CtIP
RPA
RAD51
MRE11
RAD50
Fanconi anemia pathway
BRCA2
FANCD2
FANCA
FANCC
FANCE
FANCF
FNACG
FANCL

B

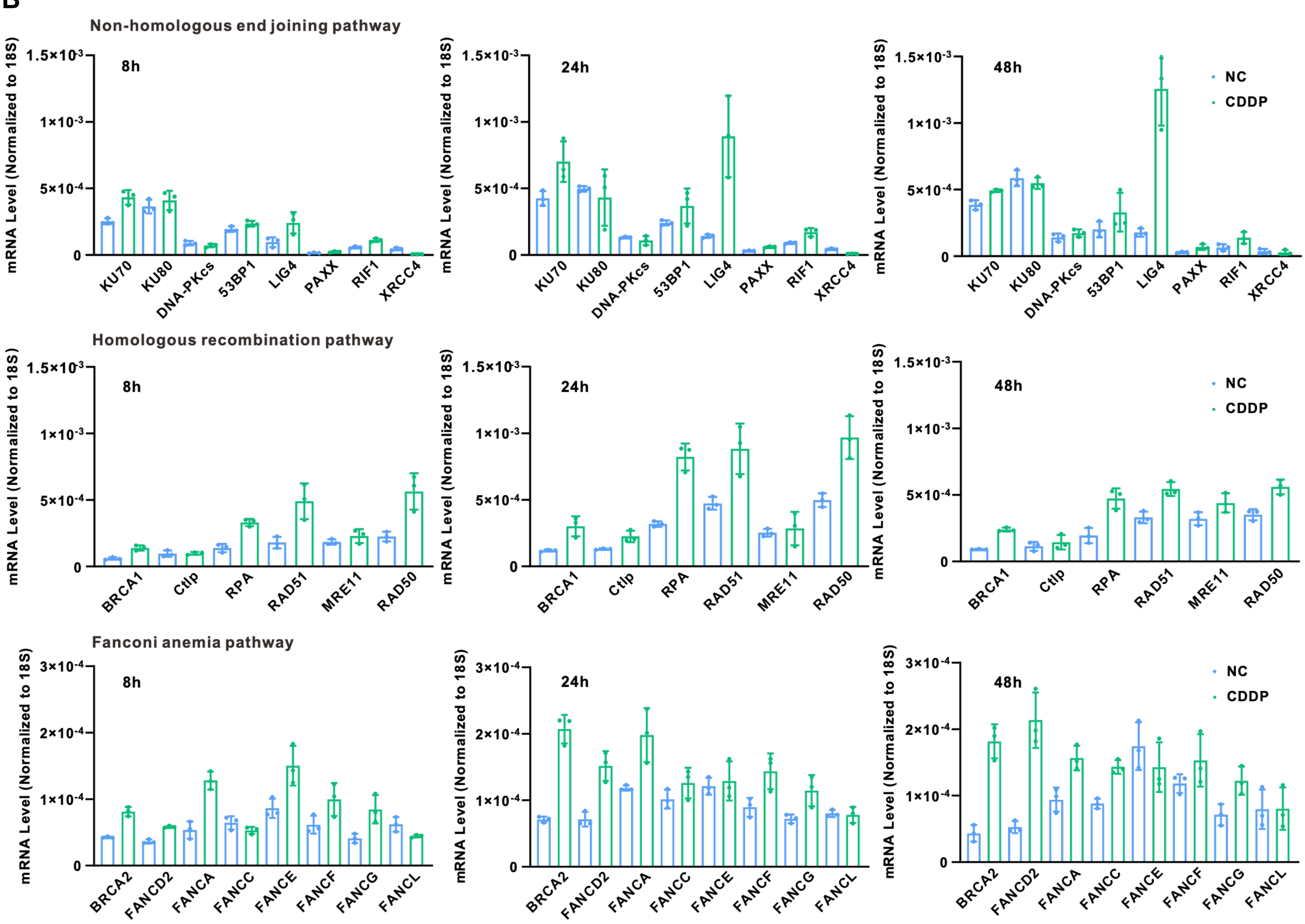

Non-homologous end joining pathway
8h
24h
48h
NC
CDDP
mRNA Level (Normalized to 18S)
KU70
KU80
DNA-PKcs
53BP1
LIG4
PAXX
RIF1
XRCC4
Homologous recombination pathway
BRCA1
CtIP
RPA
RAD51
MRE11
RAD50
Fanconi anemia pathway
BRCA2
FANCD2
FANCA
FANCC
FANCE
FANCF
FANCG
FANCL

Figure S9

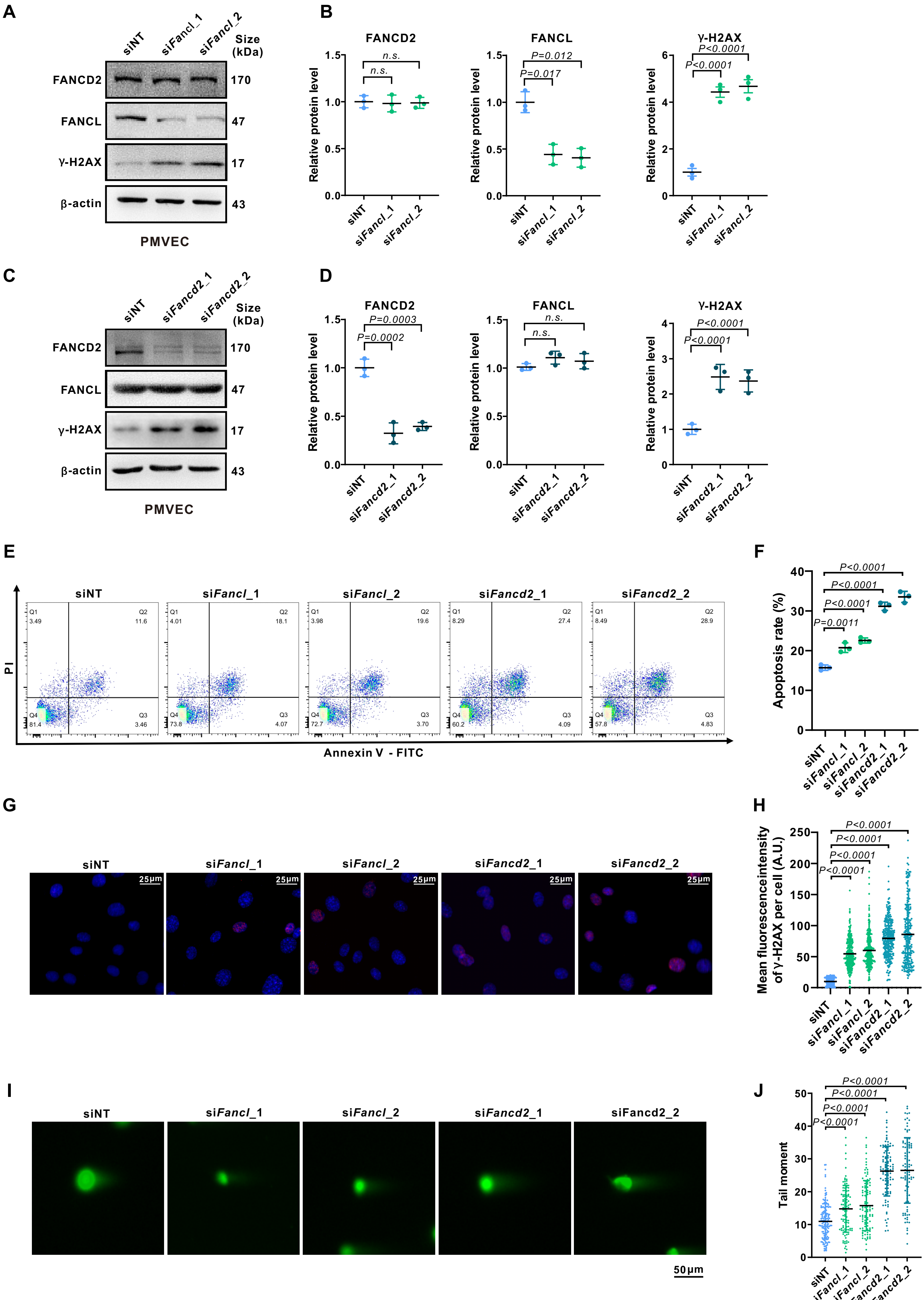
A
siNT
siFancl_1
siFancl_2
Size (kDa)
FANCD2
170
FANCL
47
γ-H2AX
17
β-actin
43
PMVEC
B
FANCD2
FANCL
γ-H2AX
Relative protein level
n.s.
P=0.012
P=0.017
P<0.0001
C
siFancd2_1
siFancd2_2
D
P=0.0003
P=0.0002
E
siNT
siFancl_1
siFancl_2
siFancd2_1
siFancd2_2
PI
Annexin V - FITC
Q1
Q2
Q3
Q4
3.49
11.6
3.46
81.4
4.01
18.1
4.07
73.8
3.98
19.6
3.70
72.7
8.29
27.4
4.09
60.2
8.49
28.9
4.83
57.8
F
Apoptosis rate (%)
P<0.0001
P=0.0011
G
25μm
H
Mean fluorescence intensity of γ-H2AX per cell (A.U.)
I
50μm
J
Tail moment

Figure S10

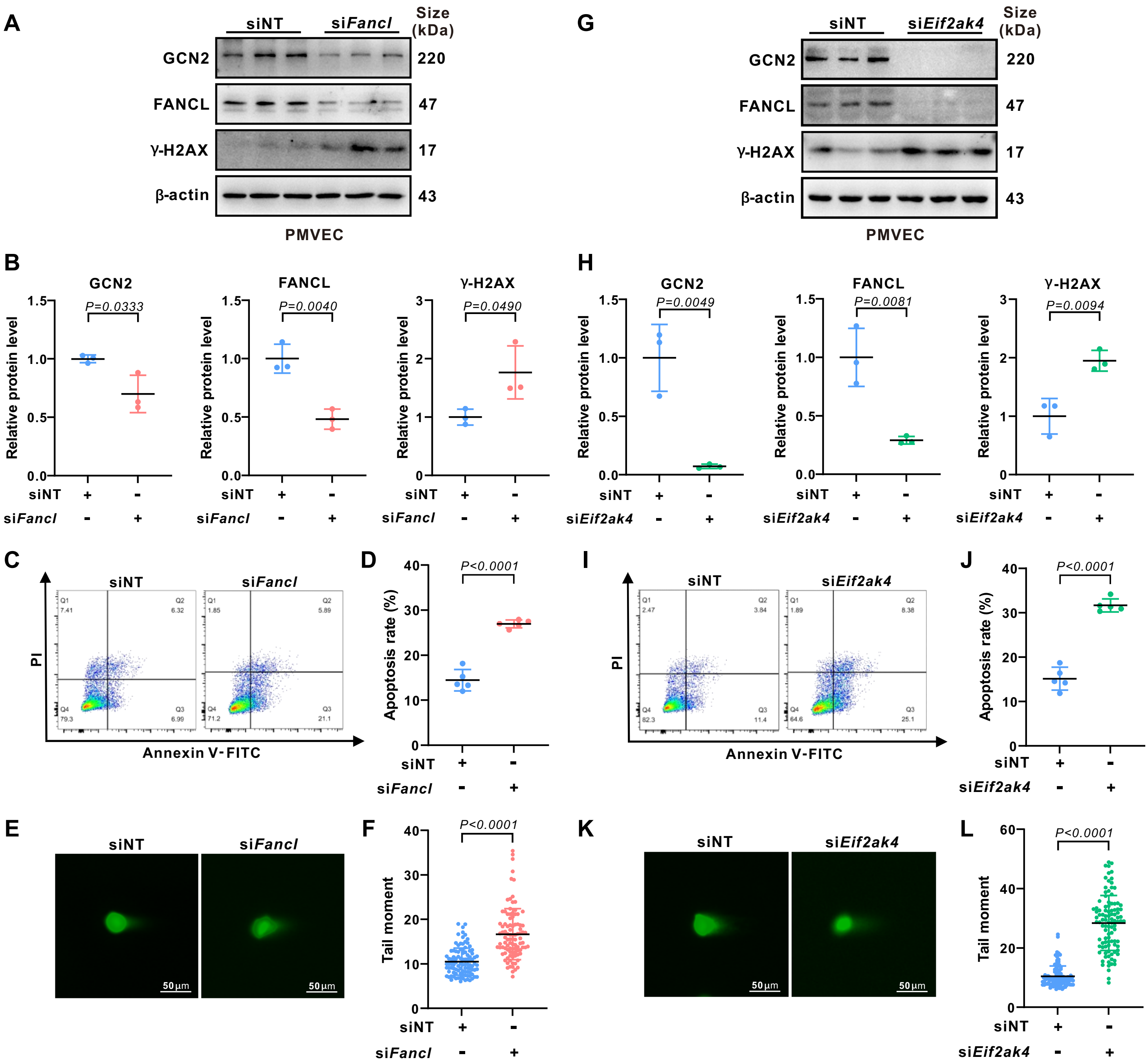
A
siNT
siFancl
Size (kDa)
GCN2
220
FANCL
47
γ-H2AX
17
β-actin
43
PMVEC
G
siNT
siEif2ak4
Size (kDa)
GCN2
220
FANCL
47
γ-H2AX
17
β-actin
43
PMVEC
B
GCN2
P=0.0333
FANCL
P=0.0040
γ-H2AX
P=0.0490
Relative protein level
siNT
siFancl
H
GCN2
P=0.0049
FANCL
P=0.0081
γ-H2AX
P=0.0094
Relative protein level
siNT
siEif2ak4
C
siNT
siFancl
PI
Annexin V-FITC
D
P<0.0001
Apoptosis rate (%)
siNT
siFancl
I
siNT
siEif2ak4
PI
Annexin V-FITC
J
P<0.0001
Apoptosis rate (%)
siNT
siEif2ak4
E
siNT
siFancl
50μm
F
P<0.0001
Tail moment
siNT
siFancl
K
siNT
siEif2ak4
50μm
L
P<0.0001
Tail moment
siNT
siEif2ak4

Figure S11

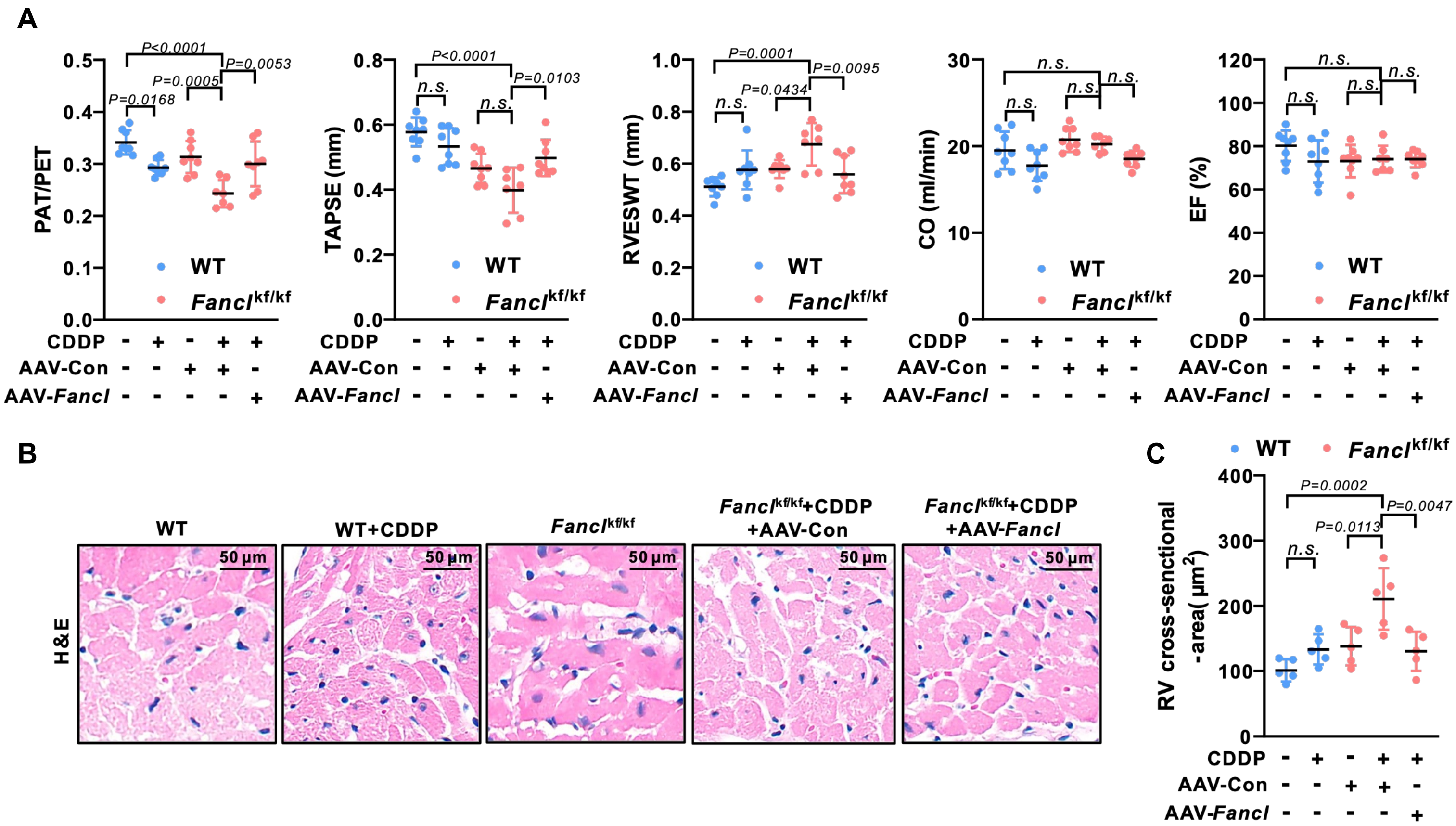
A
PAT/PET
P<0.0001
P=0.0005
P=0.0053
P=0.0168
TAPSE (mm)
P<0.0001
P=0.0103
n.s.
RVESWT (mm)
P=0.0001
P=0.0434
P=0.0095
CO (ml/min)
EF (%)
WT
Fancl kf/kf
CDDP
AAV-Con
AAV-Fancl
B
WT
WT+CDDP
Fancl kf/kf
Fancl kf/kf+CDDP +AAV-Con
Fancl kf/kf+CDDP +AAV-Fancl
H&E
50 μm
C
RV cross-senctional -area(μm2)
P=0.0002
P=0.0113
P=0.0047
n.s.

Figure S12

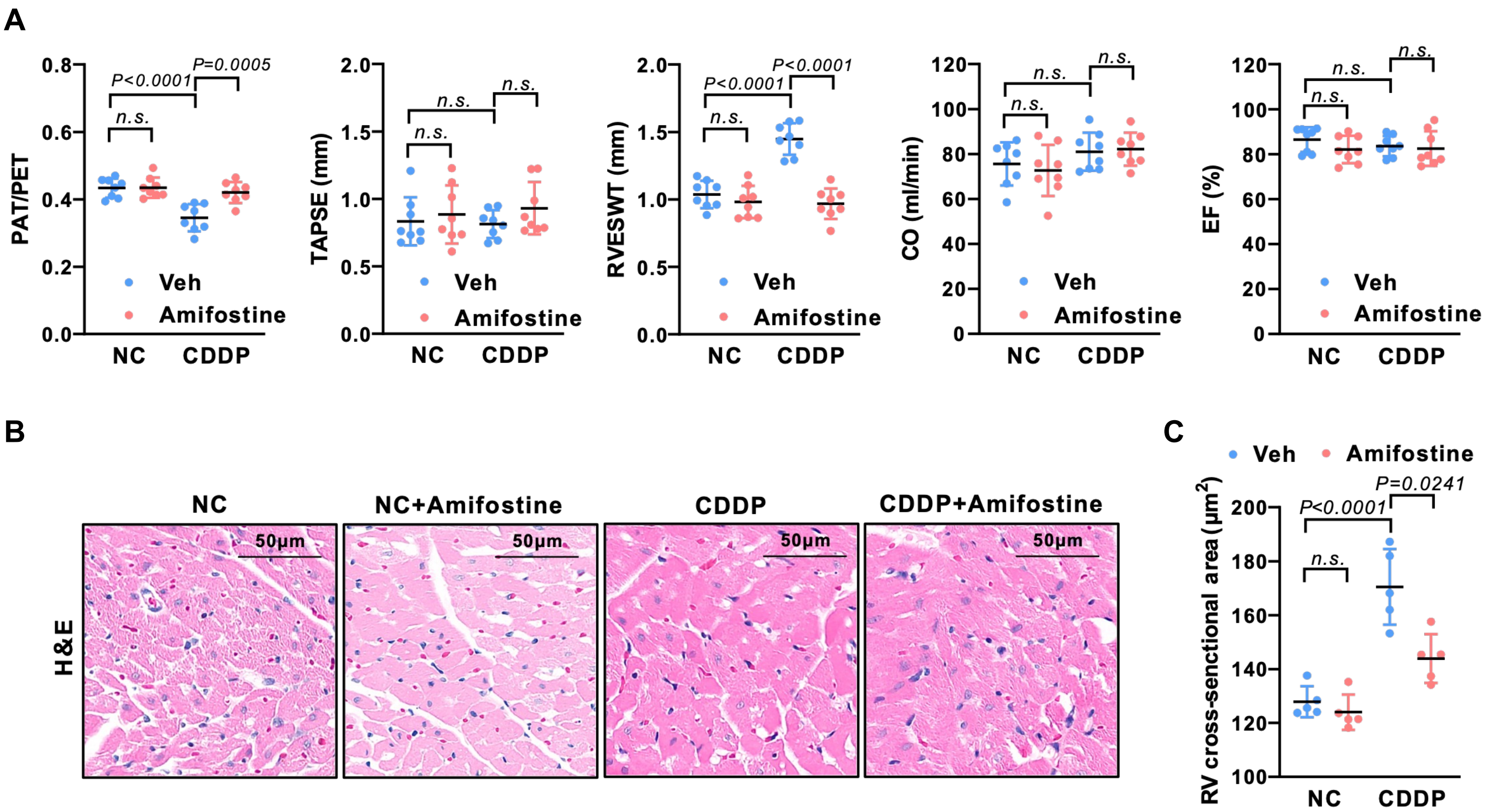

A
PAT/PET
TAPSE (mm)
RVESWT (mm)
CO (ml/min)
EF (%)
Veh
Amifostine
NC
CDDP
P<0.0001
P=0.0005
n.s.
B
NC
NC+Amifostine
CDDP
CDDP+Amifostine
H&E
50μm
C
RV cross-senctional area (μm2)
P=0.0241
