# Supplementary material for: Alkylating Agent–Induced FANCL Impairment Damages Pulmonary Arterial, Venous, and Microvascular Endothelial Cells and Predisposes to PAH/PVOD
Source: Adv Sci (Weinh). 2026 Oct 8:e78081. Online ahead of print. doi: 10.1002/advs.78081 (PMC13650023; doi:10.1002/advs.78081)
Supplement: Supplementary file 2 [file ADVS-9999-e78081-s001.docx]

**SUPPLEMENTAL MATERIAL**

**Alkylating Agent–Induced FANCL Impairment Damages Pulmonary Arterial, Venous, and Microvascular Endothelial Cells and Predisposes to PAH/PVOD**

Shiyun Liu^#^, Yufei Sun^#^, Xinlei Wu^#^, Haixia Chen^#^, Shaocong Mo, Zichong Li, Yiwei Gong, Xiaoqian Shan, Yuanqi Li, Yaru Guan, Junqi Zhu, Han Yan, Min Du, Feng Wei, Peixin Ou, Ziyi Wang, Chenting Zhang, Lei Xu, Wenju Lu, Jian Wang*, Kai Yang*

*Corresponding Authors: Kai Yang ([kyang31@gzhmu.edu.cn](mailto:kyang31@gzhmu.edu.cn)) and

Jian Wang ([jianwang@gzhmu.edu.cn](mailto:jianwang@gzhmu.edu.cn))

**This File Includes:**

Extended Methods

Figures S1–S12

Table S1

**EXTENDED METHODS**

**Ethics for Human Sample Collection**

The study protocols using human tissues and cells were approved by the ethics committee of The First Affiliated Hospital of Guangzhou Medical University in conformity with the declaration of Helsinki. The human lung tissues from patients with PVOD and donor controls were obtained from The First Affiliated Hospital of Guangzhou Medical University. Informed consent was obtained and the clinical characteristics of the PVOD patients and donor controls were provided in previous published works ^1, 2^.

**Histological, Immunofluorescent, and Immunohistochemistry Measurements of the Lung and Heart Tissues**

After anesthesia, the lung tissues were carefully perfused with phosphate buffered saline (PBS), isolated and fixed with 4% paraformaldehyde, paraffin embedded and cross sectioned at 5 μm thickness. Hematoxylin and eosin (H&E) staining was performed to visualize and quantify the vascular wall thickness of pulmonary vessels with an outer diameter (OD) between 25 μm and 50 μm. Weigert’s resorcin-fuchsin was performed to visualize elastin fibers through which distal pulmonary arteries, veins and microvessels were distinguished. Masson’s trichrome was performed to visualize and quantify the level of fibrosis in the right ventricle. Immunofluorescent (IF) staining was performed labeling DNA double strand breaks marker γH2AX, barrier junction marker ZO-1, smooth muscle cell marker α-SMA, or endothelial cell marker CD31. Immunohistochemistry (IHC) staining was performed to localize FANCL, γH2AX or Twist expression in right ventricle, or FANCD2, FANCL, γH2AX or cleaved caspase-3 in lung tissue. TUNEL assay was performed to reflect cell apoptosis in right ventricle of each group of rats. Quantitative analysis of IF intensity was performed by Image-Pro Plus 6.0.

**Fresh Isolation and Culture of Rat PMVECs**

After anesthesia, the lung lobes were isolated from each group of rats, minced and digested with 2 mg/mL of type 1 collagenase, and gently agitated for 40 minutes at 37 °C. The non-ECs were excluded by CD45 microbeads (130-052-301, Miltenyi Biotec, Bergisch Gladbach, Germany), and the ECs were enriched by CD31 microbeads (130-097-418, MiltenyiBiotec, Bergisch Gladbach, Germany) via magnetic separation according to the manufacturer’s instructions. For fresh isolation experiments, PMVECs were directly subjected to assessments of morphology, phenotype, apoptosis, IF staining and comet assay, maximally retaining the *in vivo* cell phenotype. For primary culture experiments, PMVECs isolated from control rats were serum cultured in EGM-2 Endothelial Cell Growth Medium-2 (Lonza, Walkersville, MD, USA) and treated with MMC or CDDP and then subjected to each experimental assessment.

**Isolation and Primary Culture of Rat Pulmonary Arterial, Venous, and Microvascular Endothelial Cells**

Following protocols shown in **Figure 5**, the rat PAECs, PVECs, and PMVECs were isolated using the following protocol. Intrapulmonary pulmonary arteries and veins were dissected, minced, and digested in 2 mg/mL type 1 collagenase at 37 °C for 40 min with intermittent agitation. For PMVECs, peripheral lung tissue was minced and digested under the same conditions. The resulting cell suspensions were filtered through a 70-μm cell strainer, centrifuged (500g, 10 min). The non-ECs were excluded by CD45 microbeads (130-052-301, Miltenyi Biotec, Bergisch Gladbach, Germany), and the ECs were enriched by CD31 microbeads (130-097-418, MiltenyiBiotec, Bergisch Gladbach, Germany) via magnetic separation. The isolated endothelial cells were cultured in EGM-2 Endothelial Cell Growth Medium-2 (Lonza, Walkersville, MD, USA) supplemented with 10 % fetal bovine serum and the recommended growth supplements, with medium changes every 2-3 days.

**Cell Proliferation Assay**

Cell proliferation was assessed using the Cell-Light EdU Apollo488 In Vitro Kit (RIBOBIO, Guangzhou, China) according to the manufacturer’s protocol. Briefly, cells were seeded in 96-well plates at a density of 1 × 10⁴ cells per well and cultured overnight. The cells were then incubated with 50 μM EdU for 4 h at 37 °C. After EdU labeling, cells were fixed with 4% paraformaldehyde for 30 min, permeabilized with 0.5% Triton X-100 for 10 min, and stained with Apollo488 according to the kit instructions. Nuclei were counterstained with DAPI. Fluorescence images were captured using a fluorescence microscope (DMi8, Leica, Germany). The percentage of EdU-positive cells was quantified using Image-Pro Plus 6.0 (Media Cybernetics, USA).

**Tube Formation Assay**

Tube formation capacity was assessed using Matrigel (356234, Corning, NY, USA). Briefly, 10 µL of thawed Matrigel was carefully added to each well of pre‑cooled angiogenesis culture plates (Mogengel® Cell Tube Formation Plate, 15 wells, Mogengel‑Bio, Xiamen, China) and allowed to polymerize at 37 °C for 40 min. Endothelial cells were then seeded onto the solidified Matrigel at a density of 2 × 10⁴ cells per well in EGM-2 medium. After 3-4 h of incubation at 37 °C, tube structures were visualized and imaged using a microscope scanning system (DMi1, Leica, Wetzlar, Germany). Total tube length, number of junction points and number of branch points were quantified using ImageJ software (National Institutes of Health, USA).

**Cell Apoptosis Assay**

The cell apoptosis of PMVECs from each group of rats was assessed by flow cytometry using TransDetect Annexin V-FITC/PI Cell Apoptosis Detection Kit (TransGen Biotech, Beijing, China) following the manufacturer’s instruction.

**Comet Assay**

Reheat the 1.5% low-melting agarose at 100°C until the agarose is completely dissolved. Mix gently and cool to 37°C for at least 30 min. A 10 μL of cell suspension at 1 × 10^5^ cells/mL was mixed with 90 µL of liquid pre-warmed low-melting agarose (16520050, Thermo Fisher Scientific). Mixtures were placed on a glass slide. After agarose gel solidified, the slides were dipped into a cold lysis solution (1% Triton X-100, 10% dimethyl sulfoxide and 89% lysis buffer containing 10 mM Tris, 2.5 M NaCl and 100 mM Na_2_EDTA with pH 10) for 1 h or overnight at 4°C. After lysis, agarose gels were placed in a horizontal electrophoresis chamber filled with cold buffer and incubated for 20 min in the dark for DNA unwinding and then electrophoresis was performed at 21V/cm for 40 min at 4°C. For neutralization, the slides were immersed in DNA precipitation solution (containing 7.5 M NH_4_Ac 6.7 mL and 95% EtOH 43.3 mL per 50 mL for 30 min, then in 70% ethanol for 5 min. Dry samples at 37°C for 10-15 min before being stained with nucleic acid dye. Drying brings all the cells in a single plane to facilitate observation. Slides were observed and analyzed by using Leica Dmi8 inverted microscopy. Tail moment was scored using Casplab software in at least 100 random nuclei per sample.

**Bulk RNA Sequencing (RNA‑seq) and Data Analysis**

Culture rat PMVECs were treated with MMC or CDDP for 24 h and 48 h. Total RNAs were extracted and assessed for integrity and concentration using an Agilent 2100 Bioanalyzer (Agilent Technologies, Santa Clara, CA, USA); samples with RIN > 8.0 were used for library construction. cDNA libraries were prepared with the NEBNext Ultra RNA Library Prep Kit for Illumina (E7530, NEB, Ipswich, MA, USA) and NEBNext Multiplex Oligos (E7500, NEB), and sequenced on an Illumina HiSeq platform (Biomarker Technologies, Beijing, China). Raw reads were filtered to remove adaptor sequences and low‑quality bases; clean reads were aligned to the rat reference genome (Rnor_6.0) using HISAT2. Gene expression levels were quantified as FPKM using featureCounts. Differential expression analysis was performed with DESeq2 (R package) using a negative binomial generalized linear model. Genes with |log₂(fold change)| > 1 and adjusted P < 0.05 were considered differentially expressed. Principal component analysis (PCA) was performed to assess sample clustering and group separation. Gene set enrichment analysis (GSEA) was conducted using the Hallmark gene sets to identify enriched biological pathways. Results were visualized using the ggplot2 R package.

**Real-Time qPCR**

The total RNAs were isolated from each group of cells using the Total RNA Extraction Reagent Kit (EZBioscience, EZB-TZ1). The concentration and purity of extracted RNAs were determined by optical density measurements using a Nano-Drop spectrophotometer (Thermo Fisher Scientific, Rockford, IL, USA). The HiScript II 1st Strand cDNA Synthesis Kit (Vazyme Biotech Co., Ltd., Nanjing, China) was used in a thermal cycler (Bio-Rad, Hercules, CA, USA). The PCR primers were designed from Sangon Biotech (Shanghai, China). RT-qPCR was performed on a CFX96™ Real Time System (Bio-Rad, Hercules, CA, USA) using Taq Pro Universal SYBR qPCR Master Mix (Vazyme Biotech Co., Ltd., Nanjing, China). The primer sequences for each gene are listed in **Table S1**.

**Western Blot**

The lung tissue or cultured cells were lysed in 1×RIPA buffer (GBCBIO Technologies, Guangzhou, China) containing protease inhibitor cocktail (Sigma-Aldrich, St. Louis, MO, USA) and PMSF (GBCBIO Technologies, Guangzhou, China). The expressions of specific proteins were detected by incubation of antibodies and β-actin, which serves as an internal control protein. Band intensity was quantified by ImageJ.

**SUPPLEMENTAL REFERENCE**

1. Zhang C, Lu W, Luo X, Liu S, Li Y, Zheng Q, Liu W, Wu X, Chen Y, Jiang Q, Zhang Z, Gu G, Chen J, Chen H, Liao J, Liu C, Hong C, Tang H, Sun D, Yang K, Wang J. Mitomycin c induces pulmonary vascular endothelial-to-mesenchymal transition and pulmonary veno-occlusive disease via smad3-dependent pathway in rats. *Br. J. Pharmacol*. 2021;178:217-235
2. Liu S, Shan X, Sun Y, Chen H, Feng H, Mo S, Bao C, Zhu J, Zhang Z, Wei F, Bai X, Xu R, Lai J, Luo H, Zhang C, Luo X, Jiang Q, Chen Y, Zhou Y, Tang H, Xu L, Lu W, Guo R, Liu C, Yang Z, Yuan JX, Xu X, Xu D, Wang J, Yang K. Deficient fancl predisposes to endothelial damage: A new therapeutic target for pulmonary hypertension. *Am J Respir Crit Care Med*. 2025;211:1474-1491

**SUPPLEMENTAL FIGURE LEGENDS**

**Figure S1. Echocardiographic and Right Ventricle Histological Analysis in Rats Treated with MMC or CDDP**

**A:** Summarized graphs of the echocardiographic indexes including PAT/PET, RV FAC, TAPSE, RVESWT, RVEDWT, CO, EF, and LVFS in rats treated with MMC (3 mg/kg, once weekly) for 2 or 4 weeks, n = 6 rats in each group. **B and C:** H&E images and analyzed graphs showing the RV cross-sectional area and Masson’s trichrome staining and analyzed graphs showing the level of fibrosis in rats treated with MMC (3 mg/kg, once weekly) for 2 or 4 weeks, n = 6 rats in each group. **D:** Summarized graphs of the echocardiographic indexes including PAT/PET, RV FAC, TAPSE, RVESWT, RVEDWT, CO, EF, and LVFS in rats treated with CDDP (4 mg/kg, once weekly) for 2 or 4 weeks. For each time point (2 weeks and 4 weeks), n = 5 rats for control groups and n = 10 rats for CDDP‑treated groups. **E and F:** H&E images and analyzed graphs showing the RV cross-sectional area and Masson’s trichrome staining and analyzed graphs showing the level of fibrosis in rats treated with CDDP (4 mg/kg, once weekly) for 2 or 4 weeks. For each time point (2 weeks and 4 weeks), n = 5 rats for control groups and n = 10 rats for CDDP‑treated groups. Data are presented as mean ± SD. Statistical comparisons were performed using the t‑test or the Mann‑Whitney U‑test. Specifically, The Mann–Whitney U-test was used for TAPSE, CO, EF, and FS at 2 weeks in A and for RVEDWT at 4 weeks in D. All other comparisons were performed using t-tests. A value of *P* < 0.05 is considered statistically significant. n.s. denotes not significant. Scale bars: 25 μm (panels B and E).

**Figure S2. Short‑Term Administration of MMC or CDDP Does Not Induce PAH/PVOD**

**A:** Summarized RVSP data from rats treated with MMC (3 mg/kg) or CDDP (4 mg/kg) for 2 or 5 days. n = 6 for the control group and n = 5 for other treatment groups. **B and C:** H&E images and analyzed graphs showing the pulmonary vascular thickness and vessel wall area in each group of rats, n = 6 rats in control group and 5 in other groups. **D and E:** H&E images and analyzed graphs showing the RV cross-sectional area in each group of rats, n = 5 rats in each group. **F:** IHC images showing the cleaved caspase-3 in the lung sections from each group of rats, n = 5 rats in each group. **G and H:** Immunoblotting and analyzed graphs showing the protein expression of cleaved caspase-3 in each group of rats, n = 3 rats in each group. Data are presented as mean ± SD. Statistical comparisons were performed using the t-test for the cleaved-caspase-3 protein expression analyses in H. One-way ANOVA followed by Tukey’s honestly significant difference (Tukey’s HSD) post hoc test was used for the comparisons in A, C, and E. n.s. indicates no statistical significance.

**Figure S3. Increased** **DNA Damage and Apoptosis in the Lung Tissue from PAH Rat Model Induced by Sugen/Hypoxia (SuHx)**

Immunoblots **(A)** and analyzed graphs **(B)** showing the protein expression of GCN2, FANCD2, FANCL, Bax/Bcl2 ratio, and γH2AX in the lung tissue from control and PAH rat models induced by SuHx, n = 5-7 rats in each group. Data are calculated ratio by the gray intensity of protein of interest to β-actin, normalized as fold change to the mean data of each control groups. Graph values are mean ± SD. Differences were analyzed by t-test for B (GCN2, FANCD2, FANCL, γH2AX) or Mann-Whitney U-test for B (Bax/Bcl2 ratio). A value of *P* < 0.05 is considered statistically significant, n.s. indicates no statistical significance.

**Figure S4. IHC Staining and TUNEL Assay on Right Ventricle in Rats Treated with MMC or CDDP**

**A:** Immunohistochemical staining for Twist, FANCL, and γH2AX in the right ventricle of rats treated with MMC (3 mg/kg, once weekly) or CDDP (4 mg/kg, once weekly) for 2 or 4 weeks. **B:** TUNEL staining showing the level of apoptosis in rats treated with MMC (3 mg/kg, once weekly) or CDDP (4 mg/kg, once weekly). Scale bar = 50 μm in A and 25 μm in B.

**Figure S5.** **Enhanced Apoptosis and DNA Damage in PMVECs Isolated from 4‑Week MMC‑Treated Rats**

**A–D:** Flow cytometry showing the time-course (8, 24 and 48 hours) treatment of 12 μM CDDP **(A and B)** or 0.5 μg/ml MMC **(C and D)** on apoptosis level of PMVECs freshly isolated from control (NC-PMVECs) or MMC-treated rats (MMC-PMVECs) for 4 weeks, n = 5 experiments in each group. **E–H:** Comet assay showing the effects of time-course (8, 24 and 48 hours) treatment of MMC (0.5 μg/ml) **(E and F)** or CDDP (12 μM) **(G and H)** on DNA damage in NC-PMVECs or MMC-PMVECs, n = 5 experiments in around 100 cells in each group. Graph values are mean ± SD. Differences between two groups were analyzed using the t-test for the apoptosis rate analyses in B and D and for the 8-h tail moment comparison between NC-PMVECs and MMC-PMVECs in F; the Mann-Whitney U-test was used for the remaining tail moment comparisons in F and H. *P* < 0.05 is considered statistically significant.

**Figure S6.** **Treatment of MMC or CDDP Induced Progressive Accumulation of DNA Damage and Apoptosis in Primarily Cultured Rat PMVECs**

**A–D:** Dose- and time-dependent effects of MMC **(A and B)** or CDDP **(C and D)** on cell viability, n = 5 experiments in each group. **E–H:** Immunoblots and analyzed graphs showing the dose-dependent effects of CDDP **(E and F)** or MMC **(G and H)** on γH2AX protein expression in primarily cultured rat PMVECs, n = 6 experiments in each group. **I and J:** IF staining and analyzed graphs showing the time-dependent effects of MMC (0.5 μg/ml) or CDDP (12 μM) on γH2AX levels, n = 6 experiments in around 100 cells. **K and L:** Comet assay showing the effects of time-course treatment of CDDP (12 μM) or MMC (0.5 μg/ml) on DNA damage, n = 5 experiments in around 100 cells. Graph values are mean ± SD. Differences were analyzed by t-test for B and D, or Mann-Whitney U-test for J and L. A value of *P* < 0.05 is considered statistically significant. Scale bar = 100 μm in I and 50 μm in K.

**Figure S7.** **Treatment of MMC or CDDP Induced Progressive Accumulation of DNA Damage and Dysfunction in Cultured Human PAECs**

**A and B:** Flow cytometry showing the time-course (24 and 48 hours) treatment of CDDP (12 μM) or MMC (0.5 μg/ml) on apoptosis level of cultured human PAECs, n = 3 experiments in each group. **C and D:** Comet assay showing the effects of time-course (24 and 48 hours) treatment of CDDP (12 μM) or MMC (0.5 μg/ml) on DNA damage in cultured human PAECs, n = 5 experiments involving around 100 cells in each group. **E and F:** Tube formation assay showing the effects of time-course (24 and 48 hours) treatment of CDDP (12 μM) or MMC (0.5 μg/ml) on angiogenic capacity in cultured human PAECs, n = 5 experiments in each group. **G–J:** Immunoblotting showing data for ZO-1, VEGFR2, FANCL, PCNA, Slug, Bcl2, Bax and γH2AX in human PAECs treated with MMC (0.5 μg/ml) or CDDP (12 μM) for 24 or 48 hours. **K and L:** IHC staining labeling γH2AX in the lung sections from control donor and PVOD patients, n = 5 in each group. Graph values are mean ± SD. Differences among three groups were analyzed by one-way ANOVA followed by Tukey’s HSD post hoc test for B, or Kruskal–Wallis test followed by Dunn’s multiple-comparisons test for D. Differences between two groups were analyzed by t-test for F (number of branches), H (except ZO1), J (except VEGFR2 at 24 h), and L, or Mann–Whitney U-test for F (number of junction points and total segment length), H (ZO1), and J (VEGFR2 at 24 h). A value of *P* < 0.05 is considered statistically significant, n.s. indicates no statistical significance.

**Figure S8.** **RT‑qPCR Analysis of Major DNA Repair Pathway Genes in Primary Rat PMVECs Treated with MMC or CDDP**

RT-qPCR showing the time-course (8, 24 and 48 hours) effects of MMC (0.5 μg/ml) **(A)** and CDDP (12 μM) **(B)** on the expression of genes in three classic DNA repair pathways, including the non-homologous end joining, homologous recombination and Fanconi anemia pathway in primarily cultured rat PMVECs. Graph values are mean ± SD, n = 3 experiments in each group.

**Figure S9.** **FA Pathway Impairment Leads to Elevated DNA Damage and Apoptosis in Primary Cultured Rat PMVECs**

**A–D:** Immunoblots and analyzed graphs showing the effects of knockdown of FANCL **(A and B)** or FANCD2 **(C and D)** on protein expression of γH2AX, FANCL and FANCD2. **E and F:** Flow cytometry showing the effects of knockdown of FANCL or FANCD2 on cell apoptosis, n = 3 experiments in each group. **G and H:** IF staining and analyzed graphs showing the knockdown of FANCL or FANCD2 on γH2AX levels, n = 3 experiments in around 100 cells. **I and J:** Comet assay showing the effects of knockdown of FANCL or FANCD2 on DNA damage, n = 3 experiments in around 100 cells. Graph values are mean ± SD. Differences were analyzed by one-way ANOVA with Tukey's honestly significant difference (Tukey's HSD) post hoc test for B, D, F, or Mann-Whitney U-test for H and J. A value of *P* < 0.05 is considered statistically significant, n.s. indicates no statistical significance. Scale bar = 25 μm in G and 50 μm in I.

**Figure S10. Mutual Regulation of FANCL and GCN2 in Primary Cultured Rat PMVECs**

**A and B:** Immunoblots and analyzed graphs showing the protein expression of GCN2, FANCL, and γH2AX in rat PMVECs treated with siNT or si*Fancl*, n = 3 experiments in each group. Data are calculated ratio by the gray intensity of protein of interest to β-actin, normalized as fold change to the mean data of each control group. **C and D:** Flow cytometry showing the effects of si*Fancl* on cell apoptosis in primarily cultured rat PMVECs, n = 5 experiments in each group. **E and F:** Comet assay showing the effects of si*Fancl* on cellular DNA damage in primarily cultured rat PMVECs, n = 5 experiments in around 100 cells in each group. **G and H:** Immunoblots and analyzed graphs showing the protein expression of GCN2, FANCL, and γH2AX in rat PMVECs treated with siNT or si*Eif2ak4*, n = 3 experiments in each group. Data are calculated ratio by the gray intensity of protein of interest to β-actin, normalized as fold change to the mean data of each control group. **I and J:** Flow cytometry showing the effects of si*Eif2ak4* on cell apoptosis in primarily cultured rat PMVECs, n = 5 experiments in each group. **K and L:** Comet assay showing the effects of si*Eif2ak4* on cellular DNA damage in primarily cultured rat PMVECs, n = 5 experiments in around 100 cells in each group. Graph values are mean ± SD. Differences between two groups were analyzed by t-test for B (GCN2, FANCL), D, H and J, or Mann-Whitney U-test for B (γH2AX), F and L. *P* < 0.05 is considered statistically significant. Scale bar = 50 μm in E and K.

**Figure S11. Echocardiographic and Right Ventricle Histological Analysis in FANCL Deficient and Rescue Mice**

**A:** Summarized echocardiographic parameters—including PAT/PET, TAPSE, RVESWT, CO, and EF—in WT and *Fancl*^kf/kf^ mice with or without 4‑week CDDP (3 mg/kg) treatment. Sample sizes: n = 8 per group, except for the CDDP+AAV‑Con group (n = 7). **B and C:** H&E images and analyzed graphs showing the RV cross-sectional area in each group of mice, n = 5 mice in each group. Graph values are mean ± SD. Differences among groups were analyzed by one-way ANOVA followed by Tukey’s HSD post hoc test for A and C. A value of *P* < 0.05 is considered statistically significant, n.s. indicates no statistical significance.

**Figure S12. Echocardiographic and Right Ventricular Histological Analysis in Rats Treated with CDDP and/or Amifostine**

**A:** Summarized echocardiographic parameters—including PAT/PET, TAPSE, RVESWT, CO, and EF—in each group of rats, n = 8 in each group. **B and C:** H&E images and analyzed graphs showing the RV cross-sectional area in each group of rats, n = 5 rats in each group. Graph values are mean ± SD. Differences among groups were analyzed by two-way ANOVA for PAT/PET, RVESWT, and CO in A and for RV cross-sectional area in C, with an interaction *P* = 0.0035 (PAT/PET), *P* < 0.0001 (RVESWT), and *P* < 0.0001 (CO). TAPSE and EF in A were analyzed by the Kruskal–Wallis test. A value of *P* < 0.05 is considered statistically significant, n.s. indicates no statistical significance.

**SUPPLEMENTAL TABLE**

**Table S1. Primer Pairs for RT‑qPCR and siRNA Sequences**

| Genes | Forward (5’-3’) | Reverse (5’-3’) |
| --- | --- | --- |
| RT-qPCR |  |  |
| *Ku70* (rat) | GAGTGAAGTGCTCTGGGTCT | CTCGCTTCTTCAGGTGCATC |
| *Ku80* (rat) | CCTGGTGAAGAATCCCCACT | CAAGGGCGTTATCAGTGCTC |
| *DNA-PKcs* (rat) | GCGACGTCCAGGTTATGAAC | GGTGAGTCTCCAGCTTGTCT |
| *53bp1* (rat) | AGGTGGAGGTTTGCATAGCT | CCTGTGTGTTCTCTCCGACT |
| *Lig4* (rat) | CAAAAGAGGTGAAGGGTGGC | AACCTTTACCCCAGTAGCCC |
| *Paxx* (rat) | CTGCAGGCTTTGACTCTCAG | CGAAGTCTACACCACCAGCT |
| *Rif* (rat) | CGCTGCTCTGTTGTTAGGTC | CGCTGCTCTGTTGTTAGGTC |
| *Xrcc4* (rat) | TGACGTTCAAGGCCGATTTG | GGCTCCGCTTTCTTCATCAG |
| *Brca1* (rat) | AGCTGGAGATGAAGGCAAGT | TGCATGATTCTCAGTGGGGT |
| *Ctip* (rat) | TCTCCCGTGTTTGGACCTAC | ACCTTTACCTCAGACCCAGC |
| *Rpa* (rat) | GCGCCAACCAACATTGTCTA | GACCACTTCCAGAATGTGCG |
| *Rad51* (rat) | GAGGTGGAGGTGAAGGGAAA | GTAAAGGAGCTGGGTCTGGT |
| *Mre11* (rat) | TTTTCCTACCAGCTCCAGGG | ACTCATGAAAGGGTCGTCGT |
| *Rad50* (rat) | GAGGTGGAGGTGAAGGGAAA | GTAAAGGAGCTGGGTCTGGT |
| *Brca2* (rat) | CACCAAAGTCCACACAGAGC | TCCGCGCTTGTTTCTTATCG |
| *Fancd2* (rat) | AGGACCTCACAACCCAGATG | CTCACTGAGCTCTTTCCCCA |
| *Fanca* (rat) | CTCCCCGAACTGTGAAGAGT | CCCCTGAAGAAGTGGATGGT |
| *Fancc* (rat) | CCCTCTCAGCTATGGACCTG | CCCTCTCAGCTATGGACCTG |
| *Fance* (rat) | AGACCCAAGCCAGAGACTTC | CGGAGCCTCGACTTCAGTAT |
| *Fancf* (rat) | CCTGGGAAGAGCTGTGTCTA | CCAGGAACCTCAAAATCGCC |
| *Fancg* (rat) | CCAGCTCGTTAGACAGGCTA | GCTCCCTAAACCCTTCCAGT |
| *Fancl* (rat) | ACCATGTGACAAAGCCCCTA | ACACTTGATCAGGGATGGCA |
| *18S* (rat) | GCAATTATTCCCCATGAACG | GGCCTCACTAAACCATCCAA |
| siRNA |  |  |
| *Fancl*_1 (rat) | CCUUGUGGACUUUCCUAUUTT | AAUAGGAAAGUCCACAAGGTT |
| *Fancl*_2 (rat) | GGAGCUGACCAUGUGACAATT | UUGUCACAUGGUCAGCUCCTT |
| *Fancd2*_1 (rat) | GCAUCCUUCUUAUCCCAAATT | UUUGGGAUAAGAAGGAUGCTT |
| *Fancd2*_2 (rat) | GCUGUCAUCUGUCCGUCUATT | UAGACGGACAGAUGACAGCTT |
| *Eif2ak4* (rat) | UCAUGUGAUUAAGUCAGACCCUUCA | UGAAGGGUCUGACUUAAUCACAUGA |
